# The Relationship between Occupation and Lung Cancer Incidence in the Women’s Health Initiative Observational Study

**DOI:** 10.53941/wah.2025.100010

**Published:** 2025-09-19

**Authors:** Ripon Hosain, Yvonne L. Michael, Robert B. Wallace, Rowan T. Chlebowski, David O. Garcia, Rami Nassir, Lucy F. Robinson, Rebecca A. Seguin-Fowler, Julie C. Weitlauf, Anneclaire J. De Roos

**Affiliations:** 1 Department of Environmental and Occupational Health, Dornsife School of Public Health, Drexel University, Philadelphia, PA 19104, USA; 2 Department of Epidemiology and Biostatistics, Dornsife School of Public Health, Drexel University, Philadelphia, PA 19104, USA; 3 Department of Epidemiology and Internal Medicine, University of Iowa, Iowa City, IA 52242, USA; 4 The Lundquist Institute, Torrance, CA 90502, USA; 5 Mel & Enid Zuckerman College of Public Health, University of Arizona, Tucson, AZ 85721, USA; 6 Pathology Department, School of Medicine, Umm Al-Qura University, Mecca 21421, Saudi Arabia; 7 Institute for Advancing Health through Agriculture (IHA), Department of Nutrition, College of Agriculture and Life Sciences, Texas A&M University System, College Station, TX 77841, USA; 8 Veterans Affairs Palo Alto Health Care System, Palo Alto, CA 94304, USA; 9 Department of Psychiatry and Behavioral Sciences and, by courtesy, Obstetrics and Gynecology, Stanford University School of Medicine, Stanford, CA 94305, USA

**Keywords:** lung cancer, occupation, Women’s Health Initiative (WHI), Standard Occupational Classification (SOC), smoking, high-risk occupations

## Abstract

**Background.:**

Lung cancer remains the foremost cause of cancer mortality among US women, with a notable proportion arising in never-smokers. While occupational exposures contribute to lung cancer risk, women have been underrepresented in occupational studies. Therefore, we examined the relationship between occupational titles and lung cancer incidence in women, stratified by smoking exposure.

**Methods.:**

Postmenopausal women (N = 93,676) entered the Women’s Health Initiative Observational Study prospective cohort beginning in 1993. Participants reported their three longest-held paid jobs at the study baseline and women were followed for health outcomes. Logistic regression models were used to estimate odds ratios (ORs) and 95% confidence intervals (CIs) for lung cancer incidence in association with ever working in a given occupation, as well as by employment duration (<10 years, ≥10 years), with adjustment for smoking, demographics, and lifestyle factors. Effect modification by smoking status was assessed in stratified models.

**Results.:**

Higher lung cancer risks were observed among women employed in management, sales, food service, and personal care occupations, as well as for several less common occupations, including life sciences, museum-related technical roles, and farming, construction, and production jobs. For example, ever working as Archivists, Curators, and Museum Technicians was associated with higher risk (OR = 2.55; 95% CI: 1.22–5.32), as was employment in Farming, Fishing, and Forestry occupations (OR = 1.98; 95% CI: 1.11–3.55), compared to women who never worked in those jobs. Longer-duration employment (≥10 years) as Life Scientists was also associated with elevated risk (OR = 2.31; 95% CI: 1.19–4.49). Most associations did not differ significantly by smoking, or were stronger among never-smokers than smokers, suggesting potential occupational risks independent of smoking.

**Conclusions.:**

Occupational factors may increase women’s lung cancer risk beyond smoking, necessitating targeted prevention and future research.

## 1. Introduction

Lung cancer is the leading cause of cancer-related death among women in the United States, surpassing breast, ovarian, and cervical cancers combined. While smoking is recognized as the primary risk factor for lung cancer, approximately 25% of lung cancer cases occur in individuals who have never smoked. Within these never-smoker cases, women are disproportionately represented, with a higher percentage of female lung cancer cases occurring among never-smokers (approximately 20% in the U.S. and Europe) than for male lung cancer cases (around 2–6%) [1,2]. This underscores the need to investigate potential risk factors other than smoking, particularly occupational exposures, which have been consistently linked to lung cancer in prior studies [3,4]. Identifying high-risk occupations is crucial for developing a comprehensive understanding of lung cancer etiology in women and for informing targeted prevention strategies to reduce carcinogenic exposures.

Previous studies have linked several occupations to increased lung cancer risk, including mining [3,5,6], construction [7–9], shipyard work [7,8,10], certain agricultural sectors [11–13], woodworking [14–16], professional driving [17–19], and commercial painting [20–22]. However, these occupations are typically male-dominated, limiting their relevance to women. Historically, occupational studies have underrepresented women, leaving gaps in understanding sex-specific cancer risks. While the NIH Office of Research on Women’s Health was established over three decades ago, these gaps persist [23–25]. As more women have entered male-dominated occupations over the past several decades [26], investigating how these roles affect their lung cancer risk is increasingly important. At the same time, a need remains for assessment of risks in jobs with traditionally high female representation. Previous studies conducted among women have found increased lung cancer incidence in association with occupation in service sector jobs like healthcare (e.g., nurses) [4,27,28], beauty services (e.g., hairdressers and beauticians) [17,29,30], food services [31–33], and housekeeping and cleaning [34–36]. Women may encounter distinct exposure patterns and risks due to variations in job tasks, use of personal protective equipment (PPE), and biological differences [25,37], emphasizing the need for sex-specific investigations into occupational cancer risks.

Understanding the interplay between smoking and occupation is also critical for disentangling their individual and combined contributions to lung cancer risk in women. While many lung cancer cases occur among never-smokers, it is also important to examine whether occupation independently contributes to risk or acts synergistically with smoking. Some occupational carcinogens may interact biologically with tobacco smoke to elevate lung cancer risk beyond additive effects [38–40]. Moreover, biological sex differences in toxic responses and smoking behaviors may further influence these interactions [25,37,41]. Examining potential effect modification through stratified analyses by smoking status can help determine whether occupational risks are independent of, or interact with, smoking.

This study investigated the association between occupational title and lung cancer incidence among women in the Women’s Health Initiative Observational Study (WHI-OS) cohort. We sought to identify occupations associated with elevated lung cancer risk among women and to assess whether smoking status modifies these associations.

## 2. Methods

### 2.1. Study Design and Population

This study used a prospective cohort design to investigate associations between occupational titles and lung cancer incidence [42], utilizing data from the WHI-OS, with 93,676 participants entered from 1993–1998 to investigate major chronic diseases in postmenopausal women in the United States [43]. The WHI-OS broad eligibility criteria included being postmenopausal, aged 50–79 years [43,44], willingness to provide written informed consent, intent to reside in the study area for at least three years, and health status associated with predicted survival of >3 years [45]. Baseline data collection included self-reported questionnaires and clinical assessments to capture occupation, demographics, lifestyle factors (e.g., smoking, alcohol use, physical activity), medical history, ethnicity, and anthropometric measures (e.g., height and weight). In this study of occupation and lung cancer incidence, we excluded women who reported any prior cancer diagnosis (except non-melanoma skin cancer) at the WHI baseline. We also restricted our analysis to women who reported at least one paid occupation.

### 2.2. Identification of Lung Cancer Cases

Incident lung cancer cases in WHI-OS were identified through a multi-step process. Follow-up via periodic surveys (questionnaires, telephone interviews) allowed for initial identification of incident health outcomes, including lung cancer diagnoses [45,46]. WHI staff verified these initial reports by conducting thorough medical record reviews. This centralized adjudication process involved examining pathology reports, physician notes, and other relevant documentation to confirm diagnoses based on established, pre-defined criteria. To maximize case ascertainment, additional cases were identified from death records [43,47]. This rigorous and multi-source approach has been a hallmark of WHI outcomes ascertainment, contributing to the reliable identification of incident disease across its long-term follow-up.

### 2.3. Assessment of Occupation

Occupational history was collected in the WHI-OS baseline questionnaire. Participants were first asked, “Have you ever had a job for which you were paid?” Those who responded affirmatively were asked to provide more detailed information with the following question: “What are the 3 full-time or part-time jobs that you have held the longest length of time since you were 18 years old?” and were prompted for the job title, industry, the age they started the job, and number of years they worked in the job. Job titles reported by participants were coded using the 2010 Standard Occupational Classification (SOC) system, developed by the Bureau of Labor Statistics (BLS). This system organizes occupations hierarchically based on tasks, skills, education, and required knowledge [48]. National Institute for Occupational Safety and Health (NIOSH) staff assigned unique SOC codes with up to 5-digit precision (including 2-digit, 3-digit, and 5-digit) based on the level of detail provided [49]. From the coded SOCs, we created summary variables to characterize women’s employment history for each SOC (at each level of precision), classifying participants according to whether they ever worked in the occupation (ever, never) and duration worked in the occupation (<10 years, ≥10 years), summarizing across their three reported longest-held jobs before the WHI-OS baseline. By this approach, each woman could be classified in up to 3 separate jobs over their career.

### 2.4. Statistical Analysis

All statistical analyses were conducted using SAS 9.4 software (SAS Institute Inc., Cary, NC, USA). We summarized baseline characteristics, including sociodemographics, risk factors including smoking, and family history of cancer. Categorical variables were presented as frequencies and percentages, while continuous variables were reported as means and standard deviations (SDs). We examined participant characteristics for the entire study population and separately by incident lung cancer during follow-up. Differences between lung cancer cases and non-cases were assessed using chi-square tests for categorical variables and t-tests for continuous variables. Occupational history (2-digit SOC level) for common jobs (ever held by >5% of the women) was summarized by various demographic and lifestyle factors to compare the frequency of each type of occupation among subsets of the study population.

To evaluate the association between occupation and lung cancer incidence, we applied logistic regression, comparing cancer cases to non-cases to estimate odds ratios (ORs) and corresponding 95% confidence intervals (CIs). We employed a case-control design because preliminary survival analysis of the longitudinal cohort using Cox proportional hazards regression revealed violation of the assumption of proportional hazards. Each occupation was modeled separately at the 2-digit, 3-digit, and 5-digit SOC levels. Three distinct relationships were modeled for each occupation at each level. Model 1 estimated the association between having ‘ever’ worked in the occupation and lung cancer incidence, with those who never worked in the particular occupation as the referent. Model 2 assessed the association of lung cancer incidence with employment duration in two categories (<10 years, ≥10 years), again comparing to those who never worked in the occupation as the referent. Model 3 was fitted to evaluate the trend in lung cancer incidence with employment duration, by estimating the association for women with ≥10 years duration in the occupation versus <10 years of employment as the reference group.

We included several covariates in the analysis to address potential confounding in the relationship between occupation and lung cancer incidence. We posited confounders based on known or hypothesized associations with both occupation and lung cancer; these included smoking [38], passive smoking exposure [50], age [51] (at baseline), education [52], race and Hispanic ethnicity [53,54], family history of cancer [55], alcohol consumption [56], and geographic region [53,57]. The selected covariates from the available data were age at enrollment (<55, 55–59, 60–64, 65–69, 70–74, ≥75 years), race (Black, White, Asian, American Indian/Alaska Native, Native Hawaiian/other Pacific Islander, more than 1 race), Hispanic ethnicity (yes, no), education (less than high school, high school graduate, some post-high school, college graduate or baccalaureate degree, post baccalaureate degree), U.S. region (Northeast, South, Midwest, West), smoking status (never, past, current), pack-years of smoking (never smoker, <5, 5 to <20, ≥20), lived with smoker as a child (yes, no, do not know), lived with smoker after age 18 (yes, no), worked with smoker (yes, no), alcohol intake (non-drinker, past drinker, <1 drink per month, <1 drink per week, 1 to 6 drinks per week, ≥7 drinks per week), and cancer in first-degree relative (yes, no). Missing data on covariates accounted for 7.9% of the sample. We applied the missing indicator method (MIM) to account for missing covariate data [58]. This approach allowed us to retain the full analytic sample by creating indicator variables for missing values, which were analyzed as separate categories in the regression models.

We identified ‘high-risk’ occupations among the results based on an OR point estimate ≥1.2 and at least one of the following criteria: (a) 95% CI that excluded 1.00, or (b) 95% CI width < 2.00 (calculated as the ratio of the upper to lower confidence limits), from either Model 1 (ever vs. never) or the Model 2 high-duration category (≥10 years vs. never), given at least 3 cases in each occupational exposure category of the model. These criteria were applied to identify meaningful associations with lung cancer risk whose estimates had adequately low variance. We also considered the duration-response relationship, hypothesizing that occupations causally linked to lung cancer would show a stronger association with greater duration of employment.

Because women were classified as having held up to three separate jobs and therefore could be included as exposed in multiple models, we assessed confounding of the results for our identified ‘high-risk’ occupations by other occupations. We adjusted for common 2-digit SOC groups (jobs held by ≥5% of women), one at a time, to compare the association across the adjustments for broad occupational categories.

We assessed effect modification by smoking status (ever vs. never smokers) using stratified logistic regression models. The method of Altman and Bland [59] was used to calculate a *p*-value for heterogeneity, comparing OR estimates across strata to assess whether the magnitude of the occupational association with lung cancer incidence varied by smoking status.

## 3. Results

**Figure 1.**
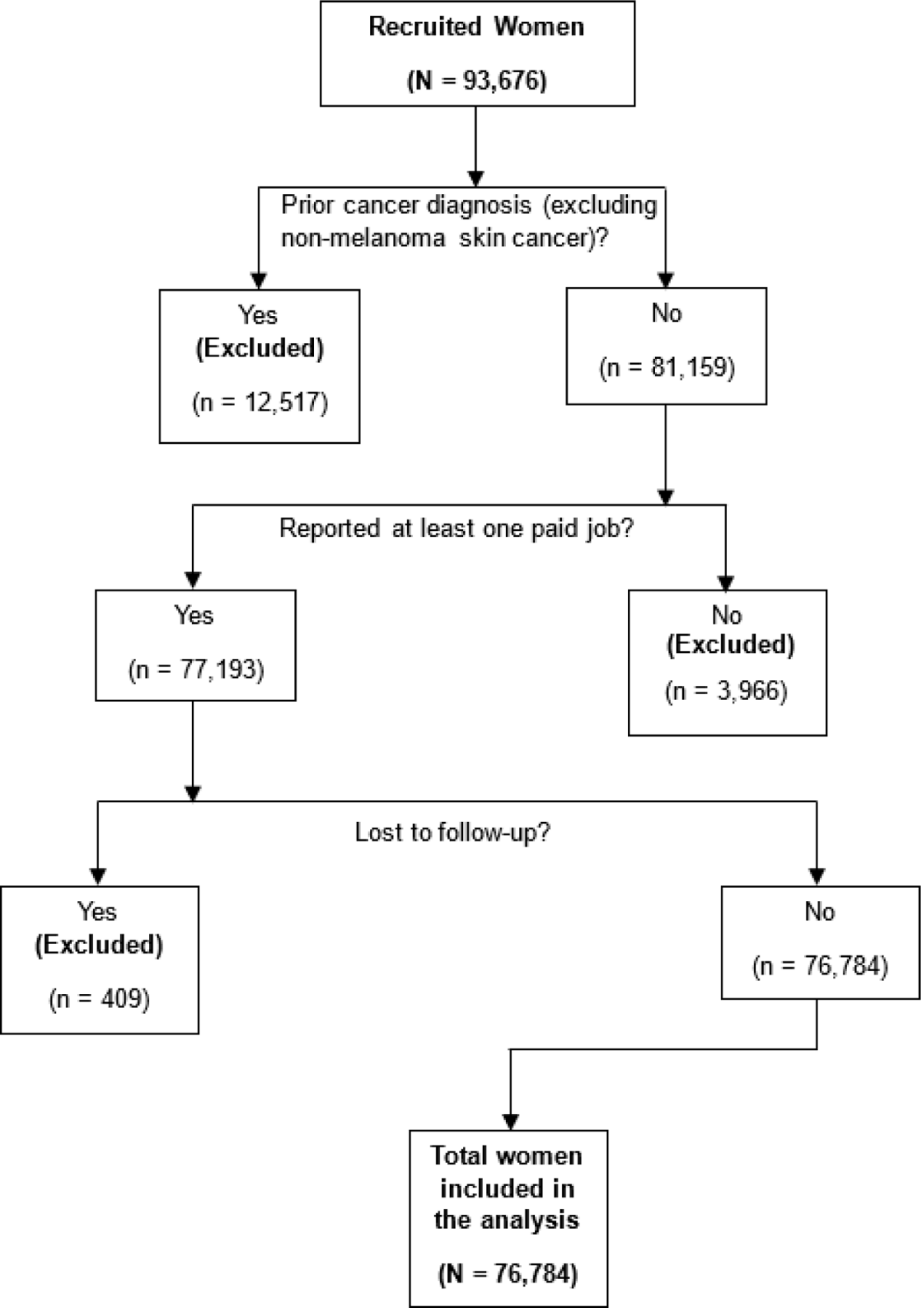
Participant inclusion in study of occupation and lung cancer incidence in the Women’s Health Initiative Observational Study.

Of the 93,676 women recruited in the WHI-OS and followed until 19 February 2023, we included a total of 76,784 women in our study. Women were excluded for the following reasons: 12,517 reported a prior cancer diagnosis (except for non-melanoma skin cancer) at baseline, 3,966 of those remaining did not report at least one paid job, and 409 were lost to follow-up (Figure 1). The 76,784 women in the study population were followed for cancer outcomes, on average, for 17.11 years (SD = 8.12 years), with a cumulative total of 1,313,774 years of follow-up.

Table 1 summarizes the characteristics of WHI-OS participants included in our study, both overall and stratified by lung cancer incidence during follow-up. Women in the study population were 63.34 years of age at the study screening (SD = 7.32), on average, and the 2075 women diagnosed with lung cancer during follow-up were typically older than the non-cases. Compared to non-cases, lung cancer cases more frequently identified as White (90.7% cases vs. 86.1% non-cases) or non-Hispanic (97.4% cases vs. 95.1% non-cases). Almost half of the women had ever smoked (past or current smokers, 48.2%), although the proportion of ever-smokers was far higher among lung cancer cases (79.9%) than non-cases (47.4%). Pack-years of smoking and passive smoking exposures were also greater among lung cancer cases. Additionally, participants who developed lung cancer were more likely to report drinking alcoholic beverages or cancer in a first-degree relative.

The most common occupations among cohort participants (ever-worked) were in Office and Administrative Support (SOC 43, 49.0%), Education, Training, and Library (SOC 25, 26.7%), and Sales and Related Occupations (SOC 41, 20.5%) (Supplementary Table S1). The youngest women at the time of the WHI study screening (<55 years) had more frequently worked in Management Occupations (SOC 11), Business and Financial Operations Occupations (SOC 13), and Community and Social Service Occupations (SOC 21), compared to older women; for example, 17.5% of women <55 years of age versus 9.6% of women 75 years or older had ever worked in Management Occupations (SOC 11). Conversely, older women had more frequently worked in Production Occupations (SOC 51). Racial and ethnic differences in occupational history were also observed. Women who self-identified as White or non-Hispanic had more frequently worked in Management (SOC 11) or Sales (SOC 41) occupations, compared to other racial groups and Hispanics, whereas Food Preparation (SOC 35) and Production (SOC 51) occupations were more frequent among Hispanic women, Black women and American Indian/Alaska Natives. Occupational history differed noticeably by smoking. Never-smokers had higher representation in Education and Library (SOC 25) occupations, whereas the percentages of women who ever worked in Management (SOC 11), Business and Financial (SOC 13), or Sales (SOC 41) occupations increased with greater pack-years of smoking. Management and Business/Financial occupations were also more common among consumers of alcohol and increased with greater frequency of alcohol consumption.

The main results for the association between occupation and lung cancer incidence in the WHI-OS cohort are presented in Table 2, which includes results for all 2-digit SOC codes in addition to identified ‘high-risk’ occupation 3-digit and 5-digit SOC codes. Supplementary Table S2 provides results from unadjusted models, while Supplementary Table S3 presents the complete set of adjusted model results corresponding to Table 2. Within Management Occupations (SOC 11), women who were ever employed as Advertising, Marketing, Promotions, Public Relations, and Sales Managers (SOC 11–2) for ≥10 years had higher lung cancer risk (OR = 1.77; 95% CI: 1.03–3.04) than those who never worked in this occupation, with evidence of a duration-response trend (*p* = 0.01). The more specific occupation of Advertising and Promotions Managers (SOC 11–201) was also associated with higher odds of lung cancer (OR = 2.58; 95% CI: 1.26–5.27). Similarly, Transportation, Storage, and Distribution Managers (SOC 11–307) had a higher incidence of lung cancer among ever-employed women (OR = 2.97; 95% CI: 1.03–8.59). Several high-risk occupations were also identified within Business and Financial Operations Occupations (SOC 13). Women who ever worked as Financial Specialists (SOC 13–2) had 20% higher incidence of lung cancer (OR = 1.20; 95% CI: 0.97–1.48), compared to women who never worked in this occupation; however, odds did not increase by duration. Among more specific jobs, elevated lung cancer incidence was observed among Financial Analysts and Advisors (SOC 13–205; ever, OR = 1.94; 95% CI: 1.13–3.33) and Credit Counselors and Loan Officers (SOC 13–207; ever, OR = 1.92; 95% CI: 1.05–3.52).

Women employed in Life, Physical, and Social Science Occupations (SOC 19) for ≥10 years experienced 40% higher lung cancer incidence (OR = 1.40; 95% CI: 1.01–1.92; p-trend duration = 0.11) than those who never worked in these occupations. Within this category, Life Scientists (SOC 19–1) had elevated odds both for ever-employed (OR = 1.81; 95% CI: 1.08–3.05) and with ≥10 years of employment (OR = 2.31; 95% CI: 1.19–4.49). Notably, Biological Scientists (SOC 19–102) exhibited the highest odds ratio in this occupational category, particularly with longer duration, based on 7 cases (≥10 years, OR = 2.62; 95% CI: 1.18–5.84; p-trend = 0.24).

In Education, Training, and Library Occupations (SOC 25), women who worked as Librarians, Curators, and Archivists (SOC 25–4) had modestly higher incidence of lung cancer (OR = 1.32; 95% CI: 0.99–1.76) than did those who never held these positions. Within this group, those employed as Archivists, Curators, and Museum Technicians (SOC 25–401) demonstrated higher incidence of lung cancer (ever, OR = 2.55; 95% CI: 1.22–5.32), although the odds did not increase substantially with longer duration (≥10 years, OR = 2.88; 95% CI: 1.02–8.11).

Personal Care and Service Occupations (SOC 39) were associated with higher lung cancer incidence (ever, OR = 1.24; 95% CI: 1.02–1.52) with no apparent trend by duration, and similar associations were seen for Other Personal Care and Service Workers (SOC 39–9; OR = 1.27; 95% CI: 0.97–1.65). Although no other occupations within this group met our predefined criteria for ‘high-risk’, several specific job titles exhibited elevated but not statistically significant and imprecise odds ratios (Supplementary Table S3). These included Nonfarm Animal Caretakers (SOC 39–202; 4 cases, OR = 2.34; 95% CI: 0.81–6.81), Tour and Travel Guides (SOC 39–701; 6 cases; OR = 1.88; 95% CI: 0.81–4.33), Childcare Workers (39–901; 33 cases; OR = 1.34; 95% CI: 0.94–1.91), and Recreation and Fitness Workers (SOC 39–903; 22 cases; OR = 1.51; 95% CI: 0.97–2.34).

Sales-related occupations showed higher lung cancer incidence for women who were ever employed as Sales Representatives, Wholesale and Manufacturing (SOC 41–4; OR = 1.52; 95% CI: 1.01–2.29), compared to women who never worked in those jobs. Among Food Preparation and Serving Occupations (SOC 35), women who ever worked as Food and Beverage Serving Workers (SOC 35–3) exhibited modestly higher lung cancer incidence (OR = 1.23; 95% CI: 1.00–1.53) than those who did not, although the elevation was limited to shorter-duration employment (<10 years, OR = 1.40; 95% CI: 1.09–1.81). Farming, Fishing, and Forestry Occupations (SOC 45) were associated with an elevated risk of lung cancer based on 13 cases who had ever worked in these jobs (OR = 1.98; 95% CI: 1.11–3.55). Within Construction and Extraction Occupations (SOC 47), a markedly high, but imprecise odds ratio was estimated for Sheet Metal Workers (SOC 47–221), with an OR of 15.36 (95% CI: 3.42–68.92), based on 3 cases.

Several elevated odds ratios were observed among Production Occupations (SOC 51) based on small numbers. For example, employment as Miscellaneous Textile, Apparel, and Furnishings Workers (SOC 51–609) for ≥10 years was associated with higher odds of lung cancer (4 cases, OR = 3.85; 95% CI: 1.27–11.63) in comparison with women who never worked in these jobs. Additionally, although not meeting our criteria for ‘high-risk’ designation, elevated but not statistically significant odds ratios were estimated (Supplementary Table S3) for Miscellaneous Assemblers and Fabricators (SOC 51–209; 19 cases; OR = 1.48; 95% CI: 0.92–2.37), Welding, Soldering and Brazing Workers (SOC 51–412; 5 cases; OR = 1.85; 95% CI: 0.73–4.67), and Miscellaneous Metal Workers and Plastic Workers (SOC 51–419; 8 cases; OR = 1.61; 95% CI: 0.77–3.37).

The associations observed with the identified ‘high-risk’ occupations were robust to adjustment by the 2-digit SOC occupational groups, one at a time (not shown in tables). For example, the associations varied in size among the adjusted models from odds ratios of 2.29–2.34 for Life Scientists (SOC 19–1, ≥10 years duration), 1.23–1.24 for Food and Beverage Serving Workers (SOC 35–3, ever), and 1.97–2.00 for Farming, Fishing, and Forestry Occupations (SOC 45, ever).

Table 3 presents adjusted associations between the identified high-risk occupations and lung cancer incidence, stratified by smoking status. Most occupational associations were consistent between never-smokers and ever-smokers, although small numbers of workers in several jobs hindered statistical comparison across smoking status. Elevated lung cancer incidence observed in association with employment as Advertising and Promotions Managers (SOC 11–201) was limited to ever-smokers, as none of the never-smoker women in this job developed lung cancer. In contrast, an inverse pattern was observed in Financial Analysts and Advisors (SOC 13–205), for which the association with lung cancer risk appeared more pronounced among never-smokers (OR = 3.79; 95% CI: 1.54–9.34) compared to ever-smokers (OR = 1.52; 95% CI: 0.78–2.95), with a p-heterogeneity of 0.11. Higher lung cancer incidence observed in association with life science occupations occurred more strongly among non-smokers, such as for those who ever-worked as Life Scientists (SOC 19–1; never-smokers, OR = 2.68, 95% CI: 1.17–6.13; ever-smokers, OR = 1.21, 95% CI: 0.59–2.51; p-heterogeneity = 0.16), as well as for longer duration working in the general category of Life, Physical, and Social Science Occupations (SOC 19, not shown in table for ≥10 years; never-smokers, OR = 2.29, 95% CI: 1.32–3.97; ever-smokers, OR = 1.12, 95% CI: 0.75–1.66; p-heterogeneity = 0.04).

## 4. Discussion

We conducted a comprehensive evaluation of the association between occupational title and lung cancer incidence among U.S. women within the WHI-OS cohort. Our findings indicate that several occupations which were common among WHI women were associated with increased incidence of lung cancer, even after adjusting for established risk factors such as smoking. Notably, we observed elevated risks among women employed as financial specialists and sales representatives, as well as jobs in food service and personal care services. We also identified increased risks for several less-frequently held occupations such as advertising and promotions managers, life scientists, jobs in farming, fishing and forestry, jobs as archivists, curators and museum technicians, and for particular construction and production occupations.

Women employed as Advertising, Marketing, Promotions, Public Relations, and Sales Managers demonstrated a pronounced increased risk of lung cancer, and we observed a clear duration-response relationship. These findings are consistent with limited prior studies that reported elevated lung cancer mortality among women in managerial roles [60] and a European case-control study identifying increased risk among Wholesale and Retail Managers [4]. We also observed a moderate association between lung cancer incidence and employment as Financial Specialists. While our study focused on occupational titles rather than causal exposures, potential contributing factors of lung cancer risks in management and financial occupations may include workplace second-hand smoke and sedentary behavior [61,62]. Furthermore, the elevated risk we identified among Transportation, Storage, and Distribution Managers aligns with earlier research on U.S. women in the transportation sector [60], where diesel exhaust is a known carcinogenic exposure [63]. We cannot rule out residual confounding by smoking, as management, business and financial operations occupations were disproportionately common among smokers, and were even more common with heavy smoking (Supplementary Table S1). Furthermore, the elevated risk for Advertising and Promotions Managers was only observed among smokers.

We found elevated risk of lung cancer for Life Scientists, especially for those with longer durations of employment. However, this observation contrasts with previous studies on female laboratory workers that reported no significant association [64], highlighting the need for further investigation to clarify potential etiologic exposures in these roles. Our finding on Librarians, Archivists, Curators, and Museum Technicians aligns with a previous case-control study from Europe that linked elevated lung cancer risk to occupations as Curators, Librarians, and Archivists [4]. Exposures to dusts and molds are common in these jobs, and some studies suggest potential links between chronic inhalation of these bioaerosols and respiratory health issues [65]. Maintenance of archives and museums can also involve exposure to pesticides, including antifungals, biocides, and insecticides used to protect susceptible paper and textile collections [66,67]. Exposure to elevated levels of formaldehyde and other volatile organic compounds (VOCs) are also common, emitted from structures (shelving, display cases) constructed for the purpose of displaying and protecting archived materials over time [68].

Food and Beverage Serving Workers showed a moderate increase in lung cancer incidence. These occupations were relatively common among WHI-OS women (3.4% of non-cases had ever-worked in the job). Elevated risks among food service workers align with prior studies, possibly attributable to environmental tobacco smoke and second-hand exposures associated with cooking [34,69]. These findings are also supported by global studies that report increased lung cancer risks among women engaged in high-temperature cooking, such as stir-frying and deep-frying, which generate carcinogenic cooking oil fumes containing polycyclic aromatic hydrocarbons (PAHs) [31,33]. Elevated lung cancer incidence found for Personal Care and Service Occupations was similar to that observed in a previous study [60]. Potential causal exposures include infectious agents in working closely with animals, children, and the public; secondhand smoke exposures in settings such as touring or in theaters, and contact with chemicals in hairdressing and cosmetology. Our findings also indicated elevated risks among Sales Representatives in Wholesale and Manufacturing, consistent with previous U.S. [60] and international studies on occupational lung cancer risks in sales roles in women [70].

Farming, Fishing, and Forestry Occupations showed strong associations with lung cancer incidence. Although some earlier research reported reduced lung cancer risks among agricultural workers,[71] several studies from the U.S. and Europe found increased risks among women due to exposures to insecticides, herbicides, diesel exhaust, organic dusts, and other environmental hazards [12,13,36]. Sheet Metal Workers also demonstrated elevated lung cancer risk. This association may reflect exposures to welding fumes, metal dusts, asbestos, or silica, as documented in prior research of construction jobs [8,10,72]. However, the wide confidence interval for this finding limits interpretation in our study. Production occupations, including Miscellaneous Textile, Apparel, and Furnishings Workers also demonstrated increased lung cancer risk among women with longer durations of employment. Interestingly, the risk we observed among female textile workers contrasts with findings from a prior case-cohort study, which reported an inverse association between textile work and lung cancer risk [73]. This discrepancy may reflect differences in occupational settings, time periods, or specific exposures within textile-related jobs. Textile workers are exposed via inhalation to dust and fibers, in addition to various chemicals used in textile finishing processes, such as dyes, solvents, formaldehyde-releasing resins, and flame retardants. Several of these substances, including formaldehyde, are established or suspected carcinogens [74].

Contrary to some prior U.S. and global studies, we observed no notable associations between employment in healthcare [4,28] or cleaning/housekeeping [34,35] occupations and lung cancer incidence. These discrepancies may reflect differences in occupational exposures, variations in safety regulations, and improved workplace conditions in the U.S., such as enhanced ventilation, reduced secondhand smoke exposure, and greater use of personal protective equipment (PPE). Demographic and socioeconomic differences, as well as variations in smoking prevalence may also explain these findings. In addition, cleaning/housekeeping jobs were rare in the cohort (<2%), limiting statistical power to assess associations with these jobs.

While a few occupations showed varied associations by lung cancer across smoking status, the majority demonstrated similar associations, indicating no evidence for effect modification by smoking. These results do not provide evidence of synergism between occupational exposures and smoking in carcinogenesis. It is important to note, however, the limited statistical power in the test for heterogeneity—particularly for uncommon jobs. Nevertheless, observations of elevated odds ratios (regardless of statistical significance) among both never- and ever- smokers, or predominantly among never-smokers support the idea that certain occupational exposures may independently contribute to development of lung cancer beyond any residual confounding by the well-established risk factor of smoking [75]. Our findings underscore the importance of considering both occupational roles and smoking behaviors when evaluating lung cancer risk among women.

A major strength of our study that enhances its contribution to understanding the relationship between occupation and lung cancer incidence is the large, well-characterized WHI-OS cohort study. WHI’s extensive data collection, including occupational histories, as well as information on demographics, lifestyle factors, and medical history allowed for adjustment for major confounders like smoking in our study. Moreover, the inclusion of a racially diverse population of postmenopausal women enhances the generalizability of the findings to a large segment of the female population. The WHI’s prospective design is a substantial strength, as collecting occupational histories at baseline, prior to cancer diagnoses, circumvented recall bias. Furthermore, the WHI’s rigorous outcome ascertainment protocols, including medical record confirmation and cancer registry linkage, minimized outcome misclassification bias.

Despite its strengths, our study has limitations that warrant consideration. Our study focused on occupational titles rather than specific workplace exposures—an approach that can identify high-risk jobs but requires further research for inference about causal factors. The WHI collected occupational data at baseline for the three longest-held jobs—falling short of a complete history and lacking information for jobs held during cohort follow-up. This limited occupational history may have missed short-term jobs with critical occupational exposures. To point, an Australian study found that occupational chemical exposures assessed by a job exposure matrix (JEM) were underestimated when based on the longest-held job versus a complete occupational history [76]. Nevertheless, validation studies have shown that a person’s single longest-held (or ‘usual’) job has good concordance with their current job [77,78] and we would expect even better representation of occupational history in our study with inclusion of three longest-held jobs. The jobs held during cohort follow-up are of limited relevance due to the average follow-up of 17 years and the expected decades-long latency of lung cancer [79–81]. We did not consider industry in our study, due to the limited reported information. Job tasks and occupational exposures may differ for workers with the same job title held in different industries—such as for a secretary in a law office versus a textile production facility. We expect that any such heterogeneity in jobs across industries is represented in our estimated effects for job titles, with the overall estimate most influenced by the typical industries in which the job is held. Finally, given that occupational histories were collected in the 1990s, changes in workplace environments and job roles since then may limit the generalizability of our findings to today’s occupational settings.

Another limitation of our study is the potential for residual confounding by smoking. While we adjusted for detailed measures of smoking status, pack-years, and multiple indicators of passive smoke exposure, the WHI data lacked finer details such as inhalation depth, quitting trajectories, cessation patterns, and the intensity of secondhand smoke exposure in occupational settings. These unmeasured variables may have influenced associations, particularly in occupations with minimal known exposure to carcinogens, such as management or financial roles. Further research with more granular smoking data and external validation is needed to clarify these relationships. Unmeasured workplace exposures may have also affected our results—in particular, those that vary considerably among people with the same job title. Such exposures may include both known and unknown carcinogens, and honing in on important exposures would require more extensive data collection from individual cohort members, focused on workplace activities and products used in their jobs. We also cannot rule out the possibility of spurious findings due to chance, given the large number of statistical comparisons in our study. We acknowledge chance as a possible explanation for any of the observed associations, noting in particular, elevated odds found in occupations with few known carcinogenic hazards. We attempted to avoid spurious findings in our identification of ‘high-risk’ occupations. We sought to avoid false positive findings by assuming a positive trend by duration and therefore not highlighting jobs that showed significant associations only with shorter duration. We also sought to avoid false negative findings through identification of high-risk occupations based on elevated ORs with adequate precision (confidence interval width), instead of only statistical significance.

While the size of the WHI cohort provided robust statistical power to detect associations for many occupational subgroups, power was greatest for the less specific 2-digit SOCs, which are sufficiently broad to include a variety of jobs (e.g., Personal Care and Service Occupations). In contrast, elevated odds ratios without statistical significance observed for numerous specific occupations (3- and 5-digit SOCs), particularly within categories like personal care and service, construction, and production likely reflect limitations in statistical power due to small numbers of women working in those roles. This tradeoff between adequate statistical power and categorization of jobs at a level of specificity that allows meaningful interpretation, emphasizes the need for further research within larger, perhaps occupation-specific cohorts to further identify occupational risks for lung cancer.

## 5. Conclusions

This cohort study of postmenopausal women in the WHI-OS reveals associations between certain occupations and increased incidence of lung cancer. Our findings underscore the complex, multifactorial nature of lung cancer risk among women, highlighting the influence of occupational titles alongside socioeconomic and lifestyle determinants. Duration-response relationships further strengthen some observed associations. These findings suggest the importance of continued research, in which detailed occupational histories are combined with exposure assessments to identify specific occupational practices and exposures that contribute to cancer risks. By identifying high-risk occupations, our research advocates for integrating occupational health into cancer prevention strategies, workplace safety improvements, and proactive surveillance to mitigate risks and protect workers.

## Supplementary Material

Tables S1-S3

The additional data and information can be downloaded at: https://media.sciltp.com/articles/others/2509190925440656/WAH-1550-SM-final.pdf.

## Figures and Tables

**Table 1.** Baseline characteristics of the WHI Observational Study cohort, overall and by lung cancer incidence during follow-up (frequencies and percentages).

Characteristic	Category	Entire Cohort (N = 76,784)	Lung Cancer Cases (N = 2075)	Non-Cases (N = 74,709)	*p*-Value *
Age at enrollment (years)	<55	10,591 (13.8)	201 (9.7)	10,390 (13.9)	<0.0001
	55–59	14,643 (19.1)	321 (15.5)	14,322 (19.2)	
	60–64	16,957 (22.1)	523 (25.2)	16,434 (22.0)	
	65–69	16,999 (22.1)	554 (26.7)	16,445 (22.0)	
	70–74	12,269 (16.0)	338 (16.3)	11,931 (16.0)	
	≥75	5325 (6.9)	138 (6.7)	5187 (6.9)	

Age at enrollment (years)	Mean (SD)	63.34 (7.32)	64.17 (6.80)	63.32 (7.33)	<0.0001

Race	American Indian/Alaska Native	239 (0.3)	6 (0.3)	233 (0.3)	<0.0001
	Asian	2245 (2.9)	33 (1.6)	2212 (3.0)	
	Native Hawaiian/Other PI	50 (0.1)	-	50 (0.1)	
	Black	5784 (7.5)	114 (5.5)	5670 (7.6)	
	White	66,237 (86.3)	1881 (90.7)	64,356 (86.1)	
	More than one race 783 (1.0)	18 (0.9)	765 (1.0)		
	Unknown/ Not reported	1446 (1.9)	23 (1.1)	1423 (1.9)	

Hispanic ethnicity	No	73,052 (95.1)	2021 (97.4)	71,031 (95.1)	<0.0001
	Yes	3003 (3.9)	33 (1.6)	2970 (4.0)	
	Missing	729 (1.0)	21 (1.0)	708 (1.0)	

Education	Less than high school	3282 (4.3)	81 (3.9)	3201 (4.3)	0.10
	High school graduate	12,175 (15.9)	303 (14.6)	11,872 (15.9)	
	Some post-high school	27,818 (36.2)	806 (38.8)	27,012 (36.2)	
	College graduate or Baccalaureate degree	9002 (11.7)	256 (12.3)	8746 (11.7)	
	Post Baccalaureate degree	23,926 (31.2)	613 (29.5)	23,313 (31.2)	
	Missing	581 (0.8)	16 (0.8)	565 (0.8)	

Region of U.S.	Northeast	17,979 (23.4)	542 (26.1)	17,437 (23.3)	0.0007
	South	19,268 (25.1)	491 (23.7)	18,777 (25.1)	
	Midwest	16,924 (22.0)	401 (19.3)	16,523 (22.1)	
	West	22,613 (29.5)	641 (30.9)	21,972 (29.4)	

Smoking status	Never	38,728 (50.4)	390 (18.8)	38,338 (51.3)	<0.0001
	Past	32,423 (42.2)	1176 (56.7)	31,247 (41.8)	
	Current	4640 (6.0)	481 (23.2)	4159 (5.6)	
	Missing	993 (1.3)	28 (1.4)	965 (1.3)	

Pack-years of smoking	Never smoker	38,728 (50.4)	390 (18.8)	38,338 (51.3)	<0.0001
	<5	11,040 (14.4)	155 (7.5)	10,885 (14.6)	
	5 to <20	10,704 (13.9)	315 (15.2)	10,389 (13.9)	
	≥20	13,589 (17.7)	1140 (54.9)	12,449 (16.7)	
	Missing	2723 (3.6)	75 (3.6)	2648 (3.5)	

Pack-years of smoking, among smokers	Mean (SD)	20.39 (21.93)	38.37 (26.89)	19.53 (21.29)	<0.0001

Passive smoking: lived with smoker as a child	No	27,135 (35.3)	589 (28.4)	26,546 (35.5)	<0.0001
	Yes	48,263 (62.9)	1452 (70.0)	46,811 (62.7)	
	Don’t know	1045 (1.4)	24 (1.2)	1021 (1.4)	
	Missing	341 (0.4)	10 (0.5)	331 (0.4)	

Passive smoking: Lived with smoker after age 18	No	20,157 (26.3)	324 (15.6)	19,833 (26.6)	<0.0001
	Yes	56,147 (73.1)	1744 (84.1)	54,403 (72.8)	
	Missing	480 (0.6)	7 (0.3)	473 (0.6)	

Passive smoking: Worked with smoker	No	18,949 (24.7)	334 (16.1)	18,615 (24.9)	<0.0001
	Yes	57,380 (74.7)	1727 (83.2)	55,653 (74.5)	
	Missing	455 (0.6)	14 (0.7)	441 (0.6)	

Alcohol intake	Nondrinker	8790 (11.5)	102 (4.9)	8688 (11.6)	<0.0001
	Past drinker	13,954 (18.2)	402 (19.4)	13,552 (18.1)	
	<1 drink per month	8925 (11.6)	213 (10.3)	8712 (11.7)	
	<1 drink per week	15,182 (19.8)	389 (18.8)	14,793 (19.8)	
	1 to 6 drinks per week	19,760 (25.7)	557 (26.8)	19,203 (25.7)	
	7+ drinks per week	9705 (12.6)	401 (19.3)	9304 (12.5)	
	Missing	468 (0.6)	11 (0.5)	457 (0.6)	

Cancer in first-degree relative	No	28083 (36.6)	680 (32.8)	27403 (36.7)	0.0004
	Yes	48632 (63.3)	1395 (67.2)	47237 (63.2)	
	Missing	69 (0.1)	-	69 (0.1)	

* *p*-values were calculated using chi-square tests to assess differences between lung cancer cases and non-cases.

**Table 2.** Association between occupation and lung cancer incidence in the WHI Observational Study cohort, for ever-worked and by duration of employment.

	Model 1			Model 2						Model 3
Occupational Titles (SOC 2010 Code)	Ever Worked (Referent: Never Worked)			Duration < 10 Years (Referent: Never Worked)			Duration ≥ 10 Years (Referent: Never Worked)			Duration Trend *p*-Value (≥10 Years vs. <10 Years)
	Cases n (%)	Non-Cases n (%)	OR (95% CI) *	Cases n (%)	Non-Cases n (%)	OR (95% CI) *	Cases n (%)	Non-Cases n (%)	OR (95% CI) *	
Management Occupations (SOC 11)	319 (15.4)	9861 (13.2)	1.09 (0.96–1.24)	122 (5.9)	3973 (5.3)	1.08 (0.89–1.31)	193 (9.3)	5790 (7.8)	1.09 (0.93–1.27)	0.93
Advertising, Marketing, Promotions, Public Relations, and Sales Managers (SOC 11–2)	20 (1.0)	605 (0.8)	1.04 (0.66–1.65)	5 (0.2)	364 (0.5)	0.48 (0.20–1.16)	15 (0.7)	239 (0.3)	**1.77 (1.03–3.04)**	0.01
Advertising and Promotions Managers (SOC 11–201)	9 (0.4)	91 (0.1)	**2.58 (1.26–5.27)**	-	-	-	-	-	-	-
Transportation Storage and Distribution Managers (SOC 11–307)	4 (0.2)	48 (0.1)	**2.97 (1.03–8.59)**	-	-	-	-	-	-	-
Business and Financial Operations Occupations (SOC 13)	193 (9.3)	5663 (7.6)	1.11 (0.95–1.29)	100 (4.8)	2839 (3.8)	1.12 (0.91–1.38)	92 (4.4)	2775 (3.7)	1.10 (0.88–1.37)	0.92
Financial Specialists (SOC 13–2)	96 (4.6)	2600 (3.5)	1.20 (0.97–1.48)	39 (1.9)	1125 (1.5)	1.17 (0.84–1.62)	56 (2.7)	1459 (2.0)	**1.21 (0.92–1.60)**	0.87
Financial Analysts and Advisors (SOC 13–205)	15 (0.7)	271 (0.4)	**1.94 (1.13–3.33)**	8 (0.4)	110 (0.1)	2.86 (1.36–6.03)	6 (0.3)	160 (0.2)	1.21 (0.52–2.79)	0.13
Credit Counselors and Loan Officers (SOC 13–207)	12 (0.6)	201 (0.3)	**1.92 (1.05–3.52)**	7 (0.3)	85 (0.1)	2.54 (1.13–5.70)	5 (0.2)	114 (0.2)	1.45 (0.58–3.65)	0.37
Computer and Mathematical Occupations (SOC 15)	46 (2.2)	1432 (1.9)	1.11 (0.82–1.50)	23 (1.1)	627 (0.8)	1.31 (0.85–2.02)	23 (1.1)	796 (1.1)	0.97 (0.63–1.48)	0.31
Architecture and Engineering Occupations (SOC 17)	30 (1.4)	813 (1.1)	1.19 (0.81–1.73)	18 (0.9)	466 (0.6)	1.31 (0.81–2.14)	12 (0.6)	339 (0.5)	1.07 (0.59–1.93)	0.59
Life, Physical, and Social Science Occupations (SOC 19)	76 (3.7)	2362 (3.2)	1.17 (0.92–1.49)	32 (1.5)	1200 (1.6)	0.95 (0.66–1.37)	43 (2.1)	1146 (1.5)	**1.40 (1.01–1.92)**	0.11
Life Scientists (SOC 19–1)	16 (0.8)	359 (0.5)	**1.81 (1.08–3.05)**	6 (0.3)	183 (0.2)	1.39 (0.61–3.19)	10 (0.5)	174 (0.2)	**2.31 (1.19–4.49)**	0.35
Biological Scientists (SOC 19–102)	10 (0.5)	211 (0.3)	1.81 (0.94–3.49)	3 (0.1)	104 (0.1)	1.11 (0.35–3.59)	7 (0.3)	106 (0.1)	**2.62 (1.18–5.84)**	0.24
Community and Social Service Occupations (SOC 21)	106 (5.1)	4173 (5.6)	0.90 (0.73–1.11)	45 (2.2)	2004 (2.7)	0.82 (0.60–1.11)	60 (2.9)	2123 (2.8)	0.97 (0.74–1.27)	0.40
Legal Occupations (SOC 23)	28 (1.3)	921 (1.2)	1.01 (0.68–1.48)	9 (0.4)	368 (0.5)	0.84 (0.43–1.64)	18 (0.9)	547 (0.7)	1.06 (0.66–1.72)	0.57
Education, Training, and Library Occupations (SOC 25)	492 (23.7)	20017 (26.8)	1.01 (0.89–1.15)	159 (7.7)	6529 (8.8)	0.97 (0.81–1.16)	329 (15.9)	13325 (17.9)	1.04 (0.90–1.20)	0.52
Librarians, Curators, and Archivists (SOC 25–4)	52 (2.5)	1629 (2.2)	**1.32 (0.99–1.76)**	21 (1.0)	599 (0.8)	1.35 (0.86–2.12)	31 (1.5)	1019 (1.4)	1.32 (0.91–1.91)	0.93
Archivists Curators and Museum Technicians (SOC 25–401)	8 (0.4)	136 (0.2)	**2.55 (1.22–5.32)**	4 (0.2)	73 (0.1)	2.29 (0.81–6.47)	4 (0.2)	63 (0.1)	**2.88 (1.02–8.11)**	0.76
Arts, Design, Entertainment, Sports, and Media Occupations (SOC 27)	121 (5.8)	3964 (5.3)	0.98 (0.81–1.19)	65 (3.1)	1923 (2.6)	1.10 (0.85–1.42)	54 (2.6)	1949 (2.6)	0.87 (0.66–1.16)	0.23
Healthcare Practitioners and Technical Occupations (SOC 29)	237 (11.4)	9524 (12.7)	0.90 (0.78–1.03)	69 (3.3)	2888 (3.9)	0.88 (0.68–1.12)	167 (8.1)	6558 (8.8)	0.91 (0.77–1.08)	0.78
Healthcare Support Occupations (SOC 31)	70 (3.4)	3144 (4.2)	0.85 (0.66–1.08)	37 (1.8)	1880 (2.5)	0.75 (0.54–1.05)	31 (1.5)	1218 (1.6)	0.96 (0.66–1.38)	0.34
Protective Service Occupations (SOC 33)	18 (0.9)	609 (0.8)	0.94 (0.58–1.53)	13 (0.6)	362 (0.5)	1.23 (0.70–2.18)	5 (0.2)	241 (0.3)	0.60 (0.24–1.48)	0.18
Food Preparation and Serving Related Occupations (SOC 35)	141 (6.8)	4304 (5.8)	1.14 (0.95–1.37)	87 (4.2)	2706 (3.6)	1.18 (0.94–1.47)	52 (2.5)	1508 (2.0)	1.09 (0.81–1.46)	0.66
Food and Beverage Serving Workers (SOC 35–3)	100 (4.8)	2514 (3.4)	**1.23 (1.00–1.53)**	70 (3.4)	1673 (2.2)	1.40 (1.09–1.81)	29 (1.4)	790 (1.1)	0.97 (0.66–1.43)	0.11
Building and Grounds Cleaning and Maintenance (SOC 37)	29 (1.4)	1502 (2.0)	0.89 (0.61–1.31)	16 (0.8)	806 (1.1)	0.89 (0.54–1.49)	11 (0.5)	653 (0.9)	0.81 (0.44–1.50)	0.81
Personal Care and Service Occupations (SOC 39)	112 (5.4)	3619 (4.8)	**1.24 (1.02–1.52)**	65 (3.1)	2084 (2.8)	1.31 (1.01–1.70)	45 (2.2)	1448 (1.9)	1.17 (0.86–1.59)	0.57
Other Personal Care and Service Workers (SOC 39–9)	61 (2.9)	2144 (2.9)	**1.27 (0.97–1.65)**	45 (2.2)	1494 (2.0)	1.30 (0.96–1.77)	15 (0.7)	589 (0.8)	1.22 (0.72–2.07)	0.84
Sales and Related Occupations (SOC 41)	465 (22.4)	15274 (20.4)	1.05 (0.94–1.17)	256 (12.3)	8892 (11.9)	1.01 (0.88–1.16)	207 (10.0)	6162 (8.3)	1.12 (0.97–1.31)	0.26
Sales Representatives, Wholesale and Manufacturing (SOC 41–4)	26 (1.3)	533 (0.7)	**1.52 (1.01–2.29)**	16 (0.8)	310 (0.4)	1.70 (1.01–2.86)	10 (0.5)	218 (0.3)	1.32 (0.69–2.55)	0.56
Office and Administrative Support Occupations (SOC 43)	1033 (49.8)	36550 (48.9)	0.97 (0.88–1.06)	497 (24.0)	17030 (22.9)	1.01 (0.90–1.13)	531 (25.7)	19206 (25.8)	0.93 (0.83–1.04)	0.19
Farming, Fishing, and Forestry Occupations (SOC 45)	13 (0.6)	250 (0.3)	**1.98 (1.11–3.55)**	9 (0.4)	132 (0.2)	2.31 (1.13–4.72)	3 (0.1)	92 (0.1)	1.61 (0.50–5.20)	0.60
Construction and Extraction Occupations (SOC 47)	16 (0.8)	299 (0.4)	1.66 (0.98–2.81)	11 (0.5)	171 (0.2)	2.05 (1.08–3.89)	5 (0.2)	123 (0.2)	1.20 (0.47–3.04)	0.35
Sheet Metal Workers (SOC 47–221)	3 (0.1)	9 (0.0)	**15.36 (3.42–68.92)**	-	-	-	-	-	-	-
Installation, Maintenance, and Repair Occupations (SOC 49)	8 (0.4)	341 (0.5)	0.76 (0.37–1.55)	-	-	-	-	-	-	-
Production Occupations (SOC 51)	143 (6.9)	5179 (6.9)	1.07 (0.89–1.28)	80 (3.9)	2868 (3.8)	1.07 (0.85–1.35)	61 (2.9)	2216 (3.0)	1.07 (0.82–1.40)	0.99
Miscellaneous Textile Apparel and Furnishings Workers (SOC 51–609)	4 (0.2)	101 (0.1)	1.68 (0.60–4.71)	-	-	-	4 (0.2)	36 (0.0)	**3.85 (1.27–11.63)**	N/A
Transportation and Material Moving Occupations (SOC 53)	45 (2.2)	1591 (2.1)	0.96 (0.70–1.30)	31 (1.5)	1013 (1.4)	1.01 (0.70–1.47)	13 (0.6)	552 (0.7)	0.81 (0.46–1.43)	0.52

* Odds ratios (ORs) and 95% confidence intervals (CIs) were derived from logistic regression models adjusted for covariates: age at enrollment, smoking status, pack years of smoking, worked with smoker, lived with smoker as a child, lived with smoker after age 18, alcohol intake, U. S. region, education, Hispanic, race, and cancer in first-degree relative. Table includes all occupations at the 2-digit SOC level, as well as 3-digit and 5-digit SOCs for identified ‘high-risk’ occupations (defined as OR ≥ 1.2 based on 3 or more lung cancer cases, and meeting at least one of the following criteria: (a) 95% CI width < 2.0; or (b) 95% CIs that excluded 1.00), from either Model 1 (ever vs. never) or the Model 2 high-duration category (≥10 years vs. never), given at least 3 cases in each occupational exposure category of the model. Estimates which met these criteria are bolded.

**Table 3.** Association between occupation and lung cancer incidence in the WHI Observational Study cohort by smoking status.

Occupational Titles (SOC 2010 Code)	Never-Smokers			Ever-Smokers			*p*-Value
	Cases n (%)	Non-Cases n (%)	OR (95% CI) *	Cases n (%)	Non-Cases n (%)	OR (95% CI) *		
Advertising, Marketing, Promotions, Public Relations, and Sales Managers (SOC 11–2)	2 (0.5)	250 (0.7)	0.74 (0.18–3.01)	18 (1.1)	349 (1.0)	1.12 (0.69–1.81)	0.60
Advertising and Promotions Managers (SOC 11–201)	-	-	-	9 (0.5)	53 (0.1)	3.13 (1.50–6.53)	-
Financial Specialists (SOC 13–2)	16 (4.1)	1288 (3.4)	1.19 (0.72–1.97)	78 (4.7)	1281 (3.6)	1.18 (0.93–1.51)	0.99
Financial Analysts and Advisors (SOC 13–205)	5 (1.3)	127 (0.3)	3.79 (1.54–9.34)	10 (0.6)	142 (0.4)	1.52 (0.78–2.95)	0.11
Credit Counselors and Loan Officers (SOC 13–207)	2 (0.5)	100 (0.3)	2.19 (0.54–8.94)	9 (0.5)	99 (0.3)	1.67 (0.83–3.37)	0.75
Life, Physical, and Social Science Occupations (SOC 19)	18 (4.6)	1186 (3.1)	1.35 (0.83–2.20)	56 (3.4)	1147 (3.2)	1.09 (0.82–1.44)	0.46
Life Scientists (SOC 19–1)	6 (1.5)	200 (0.5)	2.68 (1.17–6.13)	8 (0.5)	154 (0.4)	1.21 (0.59–2.51)	0.16
Biological Scientists (SOC 19–102)	4 (1.0)	120 (0.3)	2.91 (1.06–7.99)	5 (0.3)	89 (0.3)	1.19 (0.47–2.99)	0.20
Librarians, Curators, and Archivists (SOC 25–4)	14 (3.6)	878 (2.3)	1.41 (0.82–2.43)	38 (2.3)	733 (2.1)	1.31 (0.93–1.84)	0.83
Archivists Curators and Museum Technicians (SOC 25–401)	2 (0.5)	68 (0.2)	2.39 (0.58–9.82)	6 (0.4)	65 (0.2)	2.77 (1.17–6.57)	0.87
Food and Beverage Serving Workers (SOC 35–3)	16 (4.1)	1148 (3.0)	1.55 (0.93–2.59)	84 (5.1)	1328 (3.8)	1.18 (0.93–1.50)	0.35
Personal Care and Service Occupations (SOC 39)	22 (5.6)	1933 (5.0)	1.23 (0.80–1.90)	88 (5.3)	1642 (4.6)	1.24 (0.99–1.55)	0.98
Other Personal Care and Service Workers (SOC 39–9)	17 (4.4)	1215 (3.2)	1.50 (0.92–2.46)	44 (2.7)	905 (2.6)	1.19 (0.87–1.63)	0.45
Sales Representatives, Wholesale and Manufacturing (SOC 41–4)	3 (0.8)	244 (0.6)	1.26 (0.40–3.98)	23 (1.4)	282 (0.8)	1.60 (1.03–2.48)	0.72
Farming, Fishing, and Forestry Occupations (SOC 45)	2 (0.5)	130 (0.3)	1.85 (0.45–7.57)	11 (0.7)	116 (0.3)	2.07 (1.09–3.95)	0.89

* Odds ratios (ORs) and 95% confidence intervals (CIs) were derived from logistic regression models adjusted for covariates: age at enrollment, smoking status (past or current for ever-smokers), pack years of smoking, worked with smoker, lived with smoker as a child, lived with smoker after age 18, alcohol intake, U. S. region, education, Hispanic, race, and cancer in first-degree relative. This table presents all occupations identified as ‘high-risk’, except models with cells of 1 case are not shown.
