# Supplementary material for: The Relationship between Occupation and Lung Cancer Incidence in the Women’s Health Initiative Observational Study
Source: Work Health. Author manuscript; Available in PMC 2026 Feb 14. (PMC12904250; doi:10.53941/wah.2025.100010)
Supplement: Tables S1-S3 [file NIHMS2117996-supplement-Tables_S1-S3.pdf]

Supplementary Materials

Supplementary Table S1. Frequency of occupations (percentage of participants who ever-worked within major 2-digit SOC group) by characteristics of WHI Observational Study cohort participants*

Characteristics	Categories	Management Occupations (SOC 11)	Business and Financial Operations Occupations (SOC 13)	Community and Social Service Occupations (SOC 21)	Education, Training, and Library Occupations (SOC 25)	Arts, Design, Entertainment, Sports, and Media Occupations (SOC 27)	Healthcare Practitioners and Technical Occupations (SOC 29)	Food Preparation and Serving Related Occupations (SOC 35)	Sales and Related Occupations (SOC 41)	Office and Administrative Support Occupations (SOC 43)	Production Occupations (SOC 51)
**Overall**		**13.3%**	**7.6%**	**5.6%**	**26.7%**	**5.3%**	**12.7%**	**5.8%**	**20.5%**	**49.0%**	**6.9%**
Age at enrollment, Year	<55	17.5	9.7	7.2	27.8	5.5	13.6	5.4	19.4	43.9	5.6
	55 - 59	15.5	8.3	6.0	29.3	6.1	12.7	5.6	20.7	46.7	5.8
	60 - 64	13.9	7.4	5.5	27.4	5.0	13.0	6.3	21.0	49.2	6.6
	65 - 69	11.4	7.1	5.1	26.1	5.2	12.1	5.9	21.6	51.3	7.2
	70 - 74	10.3	6.6	5.0	23.4	5.1	12.8	5.6	19.2	52.1	8.4
	≥75	9.6	6.6	4.5	24.8	4.6	11.9	5.5	19.7	49.7	9.7
Smoking Status	Never	12.1	6.9	5.2	28.9	4.9	12.1	5.7	19.5	48.9	7.4
	Past	14.6	8.4	6.2	25.4	5.8	13.3	5.5	21.4	48.9	6.1
	Current	14.1	8.5	4.8	18.4	5.0	11.1	8.3	22.8	50.1	8.4
Pack-years of smoking	Never smoker	12.1	6.9	5.2	28.9	4.9	12.4	5.7	19.5	48.9	7.4
	<5	13.9	7.5	6.2	29.5	5.7	14.5	5.2	20.7	46.3	5.7
	5 to <20	14.1	8.5	6.2	25.0	5.4	13.4	5.6	21.3	48.8	6.5
	≥20	15.3	9.2	5.5	19.8	6.1	11.5	6.7	22.6	52.0	6.9
Alcohol intake	Nondrinker	8.7	4.7	4.4	25.7	3.2	11.3	7.3	18.7	45.4	11.2
	Past drinker	10.6	6.8	5.1	21.7	4.5	12.1	8.2	21.0	49.0	10.3
	<1 drink per month	11.7	7.4	5.4	24.5	4.0	13.6	6.2	20.2	52.7	7.7
	<1 drink per week	13.9	8.1	6.1	27.8	5.1	13.5	5.2	20.1	49.6	5.9
	1 to 6 drinks per week	15.8	8.5	6.0	29.6	6.6	13.2	4.6	20.8	49.0	4.6

	7+ drinks per week	16.5	9.1	5.8	29.4	7.4	11.9	3.9	21.8	48.0	3.8
Education	Less than high school	5.6	1.7	1.1	2.9	2.1	2.8	20.5	22.6	26.2	30.0
	High school graduate	7.1	5.1	0.9	4.4	2.3	1.9	10.6	27.1	68.4	13.3
	Some post-high school	11.7	8.1	2.1	7.9	4.1	14.6	5.4	24.1	63.6	6.8
	College graduate or Baccalaureate degree	14.8	10.7	6.2	31.3	8.0	21.6	3.0	19.2	43.1	3.2
	Post Baccalaureate degree	18.8	8.0	12.4	61.5	7.8	14.1	2.8	13.1	27.3	2.1
Race	American Indian/Alaska Native	10.9	5.0	3.4	16.3	2.1	13.4	13.4	15.1	33.1	11.3
	Asian	11.0	7.7	4.2	27.3	3.2	14.6	6.1	17.6	44.4	8.2
	Native Hawaiian/Other PI	8.0	10.0	4.0	12.0	4.0	8.0	8.0	16.0	60.0	6.0
	Black	12.5	6.2	8.3	24.5	2.0	14.1	7.2	12.5	35.7	11.0
	White	13.5	7.8	5.4	27.2	5.8	12.6	5.5	21.3	50.5	6.3
	More than one race	14.3	8.7	6.9	23.5	6.1	11.4	7.7	20.4	48.2	8.1
	Unknown/ Not reported	8.9	5.5	5.1	18.1	2.8	8.8	10.1	19.2	39.5	15.2
Hispanic Ethnicity	No	13.4	7.7	5.6	27.1	5.4	12.9	5.7	20.6	49.3	6.6
	Yes	10.6	5.8	5.0	19.5	3.3	9.2	8.1	18.9	41.8	13.9

*Includes all 2-digit SOC groups with at least 5% frequency (ever-worked) in the study population

Supplementary Table S2. Unadjusted association between occupation and lung cancer incidence in the WHI-OS cohort, for ever-worked and by duration of employment

	Model 1			Model 2						Model 3
Occupational titles (SOC 2010 code)	Ever worked (Referent: Never Worked)			Duration < 10 Years (Referent: Never Worked)			Duration ≥ 10 Years (Referent: Never Worked)			Duration trend p-value (≥ 10 years vs. < 10 years)
	Cases n (%)	Non-cases n (%)	OR (95% CI)*	Cases n (%)	Non-cases n (%)	OR (95% CI)*	Cases n (%)	Non-cases n (%)	OR (95% CI)*	
Management Occupations (SOC 11)	319 (15.4)	9861 (13.2)	1.19 (1.06 - 1.35)	122 (5.9)	3973 (5.3)	1.13 (0.94 - 1.37)	193 (9.3)	5790 (7.8)	1.23 (1.06 - 1.43)	0.48
Business and Financial Operations Occupations (SOC 13)	193 (9.3)	5663 (7.6)	1.25 (1.08 - 1.45)	100 (4.8)	2839 (3.8)	1.29 (1.05 - 1.59)	92 (4.4)	2775 (3.7)	1.22 (0.98 - 1.50)	0.68
Computer and Mathematical Occupations (SOC 15)	46 (2.2)	1432 (1.9)	1.16 (0.86 - 1.56)	23 (1.1)	627 (0.8)	1.32 (0.87 - 2.01)	23 (1.1)	796 (1.1)	1.04 (0.69 - 1.58)	0.43
Architecture and Engineering Occupations (SOC 17)	30 (1.4)	813 (1.1)	1.33 (0.92 - 1.92)	18 (0.9)	466 (0.6)	1.40 (0.87 - 2.24)	12 (0.6)	339 (0.5)	1.28 (0.72 - 2.28)	0.82
Life, Physical, and Social Science Occupations (SOC 19)	76 (3.7)	2362 (3.2)	1.16 (0.92 - 1.47)	32 (1.5)	1200 (1.6)	0.97 (0.68 - 1.37)	43 (2.1)	1146 (1.5)	1.36 (1.00 - 1.85)	0.15
Community and Social Service Occupations (SOC 21)	106 (5.1)	4173 (5.6)	0.91 (0.75 - 1.11)	45 (2.2)	2004 (2.7)	0.80 (0.60 - 1.08)	60 (2.9)	2123 (2.8)	1.01 (0.78 - 1.31)	0.25
Legal Occupations (SOC 23)	28 (1.3)	921 (1.2)	1.10 (0.75 - 1.60)	9 (0.4)	368 (0.5)	0.88 (0.45 - 1.71)	18 (0.9)	547 (0.7)	1.19 (0.74 - 1.90)	0.47
Education, Training, and Library Occupations (SOC 25)	492 (23.7)	20017 (26.8)	0.85 (0.77 - 0.94)	159 (7.7)	6529 (8.8)	0.84 (0.71 - 0.99)	329 (15.9)	13325 (17.9)	0.85 (0.76 - 0.96)	0.89

Arts, Design, Entertainment, Sports, and Media Occupations (SOC 27)	121 (5.8)	3964 (5.3)	1.11 (0.92 - 1.33)	65 (3.1)	1923 (2.6)	1.22 (0.95 - 1.57)	54 (2.6)	1949 (2.6)	1.00 (0.76 - 1.32)	0.29
Healthcare Practitioners and Technical Occupations (SOC 29)	237 (11.4)	9524 (12.7)	0.88 (0.77 - 1.01)	69 (3.3)	2888 (3.9)	0.85 (0.66 - 1.08)	167 (8.1)	6558 (8.8)	0.90 (0.77 - 1.06)	0.66
Healthcare Support Occupations (SOC 31)	70 (3.4)	3144 (4.2)	0.79 (0.62 - 1.01)	37 (1.8)	1880 (2.5)	0.70 (0.51 - 0.98)	31 (1.5)	1218 (1.6)	0.91 (0.63 - 1.30)	0.30
Protective Service Occupations (SOC 33)	18 (0.9)	609 (0.8)	1.06 (0.66 - 1.70)	13 (0.6)	362 (0.5)	1.29 (0.74 - 2.25)	5 (0.2)	241 (0.3)	0.75 (0.31 - 1.81)	0.30
Food Preparation and Serving Related Occupations (SOC 35)	141 (6.8)	4304 (5.8)	1.19 (1.00 - 1.42)	87 (4.2)	2706 (3.6)	1.17 (0.94 - 1.46)	52 (2.5)	1508 (2.0)	1.26 (0.95 - 1.66)	0.69
Building and Grounds Cleaning and Maintenance (SOC 37)	29 (1.4)	1502 (2.0)	0.69 (0.48 - 1.00)	16 (0.8)	806 (1.1)	0.71 (0.43 - 1.17)	11 (0.5)	653 (0.9)	0.60 (0.33 - 1.10)	0.68
Personal Care and Service Occupations (SOC 39)	112 (5.4)	3619 (4.8)	1.12 (0.92 - 1.36)	65 (3.1)	2084 (2.8)	1.13 (0.88 - 1.45)	45 (2.2)	1448 (1.9)	1.13 (0.83 - 1.52)	0.99
Sales and Related Occupations (SOC 41)	465 (22.4)	15274 (20.4)	1.12 (1.01 - 1.25)	256 (12.3)	8892 (11.9)	1.06 (0.93 - 1.21)	207 (10.0)	6162 (8.3)	1.24 (1.07 - 1.44)	0.10
Office and Administrative Support Occupations (SOC 43)	1033 (49.8)	36550 (48.9)	1.04 (0.95 - 1.13)	497 (24.0)	17030 (22.9)	1.07 (0.96 - 1.19)	531 (25.7)	19206 (25.8)	1.01 (0.91 - 1.13)	0.39
Farming, Fishing, and Forestry Occupations (SOC 45)	13 (0.6)	250 (0.3)	1.88 (1.07 - 3.28)	9 (0.4)	132 (0.2)	2.46 (1.25 - 4.84)	3 (0.1)	92 (0.1)	1.18 (0.37 - 3.72)	0.28
Construction and Extraction Occupations	16 (0.8)	299 (0.4)	1.93 (1.17 - 3.20)	11 (0.5)	171 (0.2)	2.32 (1.26 - 4.28)	5 (0.2)	123 (0.2)	1.47 (0.60 - 3.60)	0.41

(SOC 47)										
Installation, Maintenance, and Repair Occupations (SOC 49)	8 (0.4)	341 (0.5)	0.84 (0.42 - 1.70)	-	-	-	6 (0.3)	149 (0.2)	1.45 (0.64 - 3.28)	0.11
Production Occupations (SOC 51)	143 (6.9)	5179 (6.9)	0.99 (0.84 - 1.18)	80 (3.9)	2868 (3.8)	1.00 (0.80 - 1.26)	61 (2.9)	2216 (3.0)	0.99 (0.77 - 1.28)	0.94
Transportation and Material Moving Occupations (SOC 53)	45 (2.2)	1591 (2.1)	1.02 (0.76 - 1.37)	31 (1.5)	1013 (1.4)	1.10 (0.77 - 1.58)	13 (0.6)	552 (0.7)	0.85 (0.49 - 1.47)	0.43

*Odds ratios (ORs) and 95% confidence intervals (CIs) were derived from logistic regression models.

This table presents all occupations at the 2-digit SOC level.

Supplementary Table S3. Full set of results for association between occupation and lung cancer incidence in the WHI-OS cohort, for ever-worked and by duration of employment

(SOC 2010 Code)	Level of duration	Occupational titles	Cases n (%)	Non-cases n (%)	OR (95% CI)*
soc2d 11 Ever	-	Management Occupations - Ever	319 (15.4)	9861 (13.2)	1.09 (0.96 - 1.24)
soc2d 11 Duration Category	1	Management Occupations - Duration (1)lt10yrs, (2)ge10yrs	122 (5.9)	3973 (5.3)	1.08 (0.89 - 1.31)
soc2d 11 Duration Category	2	Management Occupations - Duration (1)lt10yrs, (2)ge10yrs	193 (9.3)	5790 (7.8)	1.09 (0.93 - 1.27)
soc2d 13 Ever	-	Business and Financial Operations Occupations - Ever	193 (9.3)	5663 (7.6)	1.11 (0.95 - 1.29)
soc2d 13 Duration Category	1	Business and Financial Operations Occupations - Duration (1)lt10yrs, (2)ge10yrs	100 (4.8)	2839 (3.8)	1.12 (0.91 - 1.38)
soc2d 13 Duration Category	2	Business and Financial Operations Occupations - Duration (1)lt10yrs, (2)ge10yrs	92 (4.4)	2775 (3.7)	1.10 (0.88 - 1.37)
soc2d 15 Ever	-	Computer and Mathematical Occupations - Ever	46 (2.2)	1432 (1.9)	1.11 (0.82 - 1.50)
soc2d 15 Duration Category	1	Computer and Mathematical Occupations - Duration (1)lt10yrs, (2)ge10yrs	23 (1.1)	627 (0.8)	1.31 (0.85 - 2.02)
soc2d 15 Duration Category	2	Computer and Mathematical Occupations - Duration (1)lt10yrs, (2)ge10yrs	23 (1.1)	796 (1.1)	0.97 (0.63 - 1.48)
soc2d 17 Ever	-	Architecture and Engineering Occupations - Ever	30 (1.4)	813 (1.1)	1.19 (0.81 - 1.73)
soc2d 17 Duration Category	1	Architecture and Engineering Occupations - Duration (1)lt10yrs, (2)ge10yrs	18 (0.9)	466 (0.6)	1.31 (0.81 - 2.14)
soc2d 17 Duration Category	2	Architecture and Engineering Occupations - Duration (1)lt10yrs, (2)ge10yrs	12 (0.6)	339 (0.5)	1.07 (0.59 - 1.93)
soc2d 19 Ever	-	Life Physical and Social Science Occupations - Ever	76 (3.7)	2362 (3.2)	1.17 (0.92 - 1.49)

soc2d 19 Duration Category	1	Life Physical and Social Science Occupations - Duration (1)lt10yrs, (2)ge10yrs	32 (1.5)	1200 (1.6)	0.95 (0.66 - 1.37)
soc2d 19 Duration Category	2	Life Physical and Social Science Occupations - Duration (1)lt10yrs, (2)ge10yrs	43 (2.1)	1146 (1.5)	1.40 (1.01 - 1.92)
soc2d 21 Ever	-	Community and Social Service Occupations - Ever	106 (5.1)	4173 (5.6)	0.90 (0.73 - 1.11)
soc2d 21 Duration Category	1	Community and Social Service Occupations - Duration (1)lt10yrs, (2)ge10yrs	45 (2.2)	2004 (2.7)	0.82 (0.60 - 1.11)
soc2d 21 Duration Category	2	Community and Social Service Occupations - Duration (1)lt10yrs, (2)ge10yrs	60 (2.9)	2123 (2.8)	0.97 (0.74 - 1.27)
soc2d 23 Ever	-	Legal Occupations - Ever	28 (1.3)	921 (1.2)	1.01 (0.68 - 1.48)
soc2d 23 Duration Category	1	Legal Occupations - Duration (1)lt10yrs, (2)ge10yrs	9 (0.4)	368 (0.5)	0.84 (0.43 - 1.64)
soc2d 23 Duration Category	2	Legal Occupations - Duration (1)lt10yrs, (2)ge10yrs	18 (0.9)	547 (0.7)	1.06 (0.66 - 1.72)
soc2d 25 Ever	-	Education Training and Library Occupations - Ever	492 (23.7)	20017 (26.8)	1.01 (0.89 - 1.15)
soc2d 25 Duration Category	1	Education Training and Library Occupations - Duration (1)lt10yrs, (2)ge10yrs	159 (7.7)	6529 (8.8)	0.97 (0.81 - 1.16)
soc2d 25 Duration Category	2	Education Training and Library Occupations - Duration (1)lt10yrs, (2)ge10yrs	329 (15.9)	13325 (17.9)	1.04 (0.90 - 1.20)
soc2d 27 Ever	-	Arts Design Entertainment Sports and Media Occupations - Ever	121 (5.8)	3964 (5.3)	0.98 (0.81 - 1.19)
soc2d 27 Duration Category	1	Arts Design Entertainment Sports and Media Occupations - Duration (1)lt10yrs, (2)ge10yrs	65 (3.1)	1923 (2.6)	1.10 (0.85 - 1.42)
soc2d 27 Duration Category	2	Arts Design Entertainment Sports and Media Occupations - Duration (1)lt10yrs, (2)ge10yrs	54 (2.6)	1949 (2.6)	0.87 (0.66 - 1.16)
soc2d 29 Ever	-	Healthcare Practitioners and Technical Occupations - Ever	237 (11.4)	9524 (12.7)	0.90 (0.78 - 1.03)
soc2d 29 Duration Category	1	Healthcare Practitioners and Technical Occupations - Duration (1)lt10yrs, (2)ge10yrs	69 (3.3)	2888 (3.9)	0.88 (0.68 - 1.12)
soc2d 29 Duration Category	2	Healthcare Practitioners and Technical Occupations - Duration (1)lt10yrs, (2)ge10yrs	167 (8.1)	6558 (8.8)	0.91 (0.77 - 1.08)
soc2d 31 Ever	-	Healthcare Support Occupations - Ever	70 (3.4)	3144 (4.2)	0.85 (0.66 - 1.08)
soc2d 31 Duration Category	1	Healthcare Support Occupations - Duration (1)lt10yrs, (2)ge10yrs	37 (1.8)	1880 (2.5)	0.75 (0.54 - 1.05)
soc2d 31 Duration Category	2	Healthcare Support Occupations - Duration (1)lt10yrs, (2)ge10yrs	31 (1.5)	1218 (1.6)	0.96 (0.66 - 1.38)
soc2d 33 Ever	-	Protective Service Occupations - Ever	18 (0.9)	609 (0.8)	0.94 (0.58 - 1.53)
soc2d 33 Duration Category	1	Protective Service Occupations - Duration (1)lt10yrs, (2)ge10yrs	13 (0.6)	362 (0.5)	1.23 (0.70 - 2.18)
soc2d 33 Duration Category	2	Protective Service Occupations - Duration (1)lt10yrs, (2)ge10yrs	5 (0.2)	241 (0.3)	0.60 (0.24 - 1.48)
soc2d 35 Ever	-	Food Preparation and Serving Related Occupations - Ever	141 (6.8)	4304 (5.8)	1.14 (0.95 - 1.37)
soc2d 35 Duration Category	1	Food Preparation and Serving Related Occupations - Duration (1)lt10yrs, (2)ge10yrs	87 (4.2)	2706 (3.6)	1.18 (0.94 - 1.47)
soc2d 35 Duration Category	2	Food Preparation and Serving Related Occupations - Duration (1)lt10yrs, (2)ge10yrs	52 (2.5)	1508 (2.0)	1.09 (0.81 - 1.46)
soc2d 37 Ever	-	Building and Grounds Cleaning and Maintenance Occupations - Ever	29 (1.4)	1502 (2.0)	0.89 (0.61 - 1.31)

soc2d 37 Duration Category	1	Building and Grounds Cleaning and Maintenance Occupations - Duration (1)lt10yrs, (2)ge10yr	16 (0.8)	806 (1.1)	0.89 (0.54 - 1.49)
soc2d 37 Duration Category	2	Building and Grounds Cleaning and Maintenance Occupations - Duration (1)lt10yrs, (2)ge10yr	11 (0.5)	653 (0.9)	0.81 (0.44 - 1.50)
soc2d 39 Ever	-	Personal Care and Service Occupations - Ever	112 (5.4)	3619 (4.8)	1.24 (1.02 - 1.52)
soc2d 39 Duration Category	1	Personal Care and Service Occupations - Duration (1)lt10yrs, (2)ge10yrs	65 (3.1)	2084 (2.8)	1.31 (1.01 - 1.70)
soc2d 39 Duration Category	2	Personal Care and Service Occupations - Duration (1)lt10yrs, (2)ge10yrs	45 (2.2)	1448 (1.9)	1.17 (0.86 - 1.59)
soc2d 41 Ever	-	Sales and Related Occupations - Ever	465 (22.4)	15274 (20.4)	1.05 (0.94 - 1.17)
soc2d 41 Duration Category	1	Sales and Related Occupations - Duration (1)lt10yrs, (2)ge10yrs	256 (12.3)	8892 (11.9)	1.01 (0.88 - 1.16)
soc2d 41 Duration Category	2	Sales and Related Occupations - Duration (1)lt10yrs, (2)ge10yrs	207 (10.0)	6162 (8.3)	1.12 (0.97 - 1.31)
soc2d 43 Ever	-	Office and Administrative Support Occupations - Ever	1033 (49.8)	36550 (48.9)	0.97 (0.88 - 1.06)
soc2d 43 Duration Category	1	Office and Administrative Support Occupations - Duration (1)lt10yrs, (2)ge10yrs	497 (24.0)	17030 (22.9)	1.01 (0.90 - 1.13)
soc2d 43 Duration Category	2	Office and Administrative Support Occupations - Duration (1)lt10yrs, (2)ge10yrs	531 (25.7)	19206 (25.8)	0.93 (0.83 - 1.04)
soc2d 45 Ever	-	Farming Fishing and Forestry Occupations - Ever	13 (0.6)	250 (0.3)	1.98 (1.11 - 3.55)
soc2d 45 Duration Category	1	Farming Fishing and Forestry Occupations - Duration (1)lt10yrs, (2)ge10yrs	9 (0.4)	132 (0.2)	2.31 (1.13 - 4.72)
soc2d 45 Duration Category	2	Farming Fishing and Forestry Occupations - Duration (1)lt10yrs, (2)ge10yrs	3 (0.1)	92 (0.1)	1.61 (0.50 - 5.20)
soc2d 47 Ever	-	Construction and Extraction Occupations - Ever	16 (0.8)	299 (0.4)	1.66 (0.98 - 2.81)
soc2d 47 Duration Category	1	Construction and Extraction Occupations - Duration (1)lt10yrs, (2)ge10yrs	11 (0.5)	171 (0.2)	2.05 (1.08 - 3.89)
soc2d 47 Duration Category	2	Construction and Extraction Occupations - Duration (1)lt10yrs, (2)ge10yrs	5 (0.2)	123 (0.2)	1.20 (0.47 - 3.04)
soc2d 49 Ever	-	Installation Maintenance and Repair Occupations - Ever	8 (0.4)	341 (0.5)	0.76 (0.37 - 1.55)
soc2d 51 Ever	-	Production Occupations - Ever	143 (6.9)	5179 (6.9)	1.07 (0.89 - 1.28)
soc2d 51 Duration Category	1	Production Occupations - Duration (1)lt10yrs, (2)ge10yrs	80 (3.9)	2868 (3.8)	1.07 (0.85 - 1.35)
soc2d 51 Duration Category	2	Production Occupations - Duration (1)lt10yrs, (2)ge10yrs	61 (2.9)	2216 (3.0)	1.07 (0.82 - 1.40)
soc2d 53 Ever	-	Transportation and Material Moving Occupations - Ever	45 (2.2)	1591 (2.1)	0.96 (0.70 - 1.30)
soc2d 53 Duration Category	1	Transportation and Material Moving Occupations - Duration (1)lt10yrs, (2)ge10yrs	31 (1.5)	1013 (1.4)	1.01 (0.70 - 1.47)
soc2d 53 Duration Category	2	Transportation and Material Moving Occupations - Duration (1)lt10yrs, (2)ge10yrs	13 (0.6)	552 (0.7)	0.81 (0.46 - 1.43)
soc3d 11 10 Ever	-	Top Executives - Ever	42 (2.0)	1194 (1.6)	1.12 (0.81 - 1.54)
soc3d 11 10 Duration Category	1	Top Executives - Duration (1)lt10yrs, (2)ge10yrs	16 (0.8)	581 (0.8)	0.92 (0.55 - 1.53)
soc3d 11 10 Duration Category	2	Top Executives - Duration (1)lt10yrs, (2)ge10yrs	24 (1.2)	605 (0.8)	1.19 (0.78 - 1.82)

soc3d 11 20 Ever	-	Advertising Marketing Promotions Public Relations and Sales Managers - Ever	20 (1.0)	605 (0.8)	1.04 (0.66 - 1.65)
soc3d 11 20 Duration Category	1	Advertising Marketing Promotions Public Relations and Sales Managers - Duration (1)lt10yrs	5 (0.2)	364 (0.5)	0.48 (0.20 - 1.16)
soc3d 11 20 Duration Category	2	Advertising Marketing Promotions Public Relations and Sales Managers - Duration (1)lt10yrs	15 (0.7)	239 (0.3)	1.77 (1.03 - 3.04)
soc3d 11 30 Ever	-	Operations Specialties Managers - Ever	48 (2.3)	1322 (1.8)	1.18 (0.87 - 1.59)
soc3d 11 30 Duration Category	1	Operations Specialties Managers - Duration (1)lt10yrs, (2)ge10yrs	23 (1.1)	552 (0.7)	1.40 (0.91 - 2.16)
soc3d 11 30 Duration Category	2	Operations Specialties Managers - Duration (1)lt10yrs, (2)ge10yrs	25 (1.2)	759 (1.0)	1.03 (0.68 - 1.55)
soc3d 11 90 Ever	-	Other Management Occupations - Ever	225 (10.8)	7371 (9.9)	1.03 (0.89 - 1.19)
soc3d 11 90 Duration Category	1	Other Management Occupations - Duration (1)lt10yrs, (2)ge10yrs	94 (4.5)	3160 (4.2)	1.02 (0.83 - 1.27)
soc3d 11 90 Duration Category	2	Other Management Occupations - Duration (1)lt10yrs, (2)ge10yrs	129 (6.2)	4128 (5.5)	1.04 (0.86 - 1.25)
soc3d 13 10 Ever	-	Business Operations Specialists - Ever	97 (4.7)	3151 (4.2)	0.99 (0.80 - 1.23)
soc3d 13 10 Duration Category	1	Business Operations Specialists - Duration (1)lt10yrs, (2)ge10yrs	61 (2.9)	1805 (2.4)	1.03 (0.79 - 1.35)
soc3d 13 10 Duration Category	2	Business Operations Specialists - Duration (1)lt10yrs, (2)ge10yrs	36 (1.7)	1313 (1.8)	0.95 (0.68 - 1.34)
soc3d 13 20 Ever	-	Financial Specialists - Ever	96 (4.6)	2600 (3.5)	1.20 (0.97 - 1.48)
soc3d 13 20 Duration Category	1	Financial Specialists - Duration (1)lt10yrs, (2)ge10yrs	39 (1.9)	1125 (1.5)	1.17 (0.84 - 1.62)
soc3d 13 20 Duration Category	2	Financial Specialists - Duration (1)lt10yrs, (2)ge10yrs	56 (2.7)	1459 (2.0)	1.21 (0.92 - 1.60)
soc3d 15 11 Ever	-	Computer Occupations - Ever	29 (1.4)	1026 (1.4)	0.96 (0.66 - 1.41)
soc3d 15 11 Duration Category	1	Computer Occupations - Duration (1)lt10yrs, (2)ge10yrs	10 (0.5)	420 (0.6)	0.83 (0.44 - 1.58)
soc3d 15 11 Duration Category	2	Computer Occupations - Duration (1)lt10yrs, (2)ge10yrs	19 (0.9)	601 (0.8)	1.06 (0.66 - 1.70)
soc3d 15 20 Ever	-	Mathematical Science Occupations - Ever	20 (1.0)	457 (0.6)	1.49 (0.94 - 2.37)
soc3d 15 20 Duration Category	1	Mathematical Science Occupations - Duration (1)lt10yrs, (2)ge10yrs	14 (0.7)	256 (0.3)	1.94 (1.11 - 3.39)
soc3d 15 20 Duration Category	2	Mathematical Science Occupations - Duration (1)lt10yrs, (2)ge10yrs	6 (0.3)	197 (0.3)	0.97 (0.42 - 2.23)
soc3d 17 20 Ever	-	Engineers - Ever	10 (0.5)	214 (0.3)	1.39 (0.72 - 2.68)
soc3d 17 20 Duration Category	1	Engineers - Duration (1)lt10yrs, (2)ge10yrs	3 (0.1)	101 (0.1)	0.94 (0.29 - 3.03)
soc3d 17 20 Duration Category	2	Engineers - Duration (1)lt10yrs, (2)ge10yrs	7 (0.3)	110 (0.1)	1.79 (0.81 - 3.98)
soc3d 17 30 Ever	-	Drafters Engineering Technicians and Mapping Technicians - Ever	19 (0.9)	514 (0.7)	1.17 (0.73 - 1.88)
soc3d 17 30 Duration Category	1	Drafters Engineering Technicians and Mapping Technicians - Duration (1)lt10yrs, (2)ge10yrs	14 (0.7)	328 (0.4)	1.36 (0.79 - 2.37)
soc3d 17 30 Duration Category	2	Drafters Engineering Technicians and Mapping Technicians - Duration (1)lt10yrs, (2)ge10yrs	5 (0.2)	183 (0.2)	0.87 (0.35 - 2.15)
soc3d 19 10 Ever	-	Life Scientists - Ever	16 (0.8)	359 (0.5)	1.81 (1.08 - 3.05)
soc3d 19 10 Duration Category	1	Life Scientists - Duration (1)lt10yrs, (2)ge10yrs	6 (0.3)	183 (0.2)	1.39 (0.61 - 3.19)

soc3d 19 10 Duration Category	2	Life Scientists - Duration (1)lt10yrs, (2)ge10yrs	10 (0.5)	174 (0.2)	2.31 (1.19 - 4.49)
soc3d 19 20 Ever	-	Physical Scientists - Ever	15 (0.7)	489 (0.7)	1.17 (0.69 - 1.99)
soc3d 19 20 Duration Category	1	Physical Scientists - Duration (1)lt10yrs, (2)ge10yrs	8 (0.4)	283 (0.4)	0.98 (0.47 - 2.01)
soc3d 19 20 Duration Category	2	Physical Scientists - Duration (1)lt10yrs, (2)ge10yrs	6 (0.3)	198 (0.3)	1.38 (0.60 - 3.18)
soc3d 19 30 Ever	-	Social Scientists and Related Workers - Ever	26 (1.3)	776 (1.0)	1.09 (0.73 - 1.64)
soc3d 19 30 Duration Category	1	Social Scientists and Related Workers - Duration (1)lt10yrs, (2)ge10yrs	8 (0.4)	243 (0.3)	1.04 (0.50 - 2.14)
soc3d 19 30 Duration Category	2	Social Scientists and Related Workers - Duration (1)lt10yrs, (2)ge10yrs	18 (0.9)	527 (0.7)	1.13 (0.69 - 1.83)
soc3d 19 40 Ever	-	Life Physical and Social Science Technicians - Ever	24 (1.2)	859 (1.1)	1.06 (0.70 - 1.60)
soc3d 19 40 Duration Category	1	Life Physical and Social Science Technicians - Duration (1)lt10yrs, (2)ge10yrs	16 (0.8)	610 (0.8)	1.00 (0.60 - 1.66)
soc3d 19 40 Duration Category	2	Life Physical and Social Science Technicians - Duration (1)lt10yrs, (2)ge10yrs	8 (0.4)	247 (0.3)	1.21 (0.59 - 2.49)
soc3d 21 10 Ever	-	Counselors Social Workers and Other Community and Social Service Specialists - Ever	97 (4.7)	3750 (5.0)	0.89 (0.72 - 1.10)
soc3d 21 10 Duration Category	1	Counselors Social Workers and Other Community and Social Service Specialists - Duration (1	41 (2.0)	1819 (2.4)	0.80 (0.58 - 1.11)
soc3d 21 10 Duration Category	2	Counselors Social Workers and Other Community and Social Service Specialists - Duration (1	55 (2.7)	1891 (2.5)	0.96 (0.72 - 1.27)
soc3d 21 20 Ever	-	Religious Workers - Ever	9 (0.4)	466 (0.6)	0.96 (0.49 - 1.88)
soc3d 21 20 Duration Category	1	Religious Workers - Duration (1)lt10yrs, (2)ge10yrs	4 (0.2)	226 (0.3)	0.83 (0.30 - 2.26)
soc3d 21 20 Duration Category	2	Religious Workers - Duration (1)lt10yrs, (2)ge10yrs	5 (0.2)	232 (0.3)	1.14 (0.46 - 2.81)
soc3d 23 10 Ever	-	Lawyers Judges and Related Workers - Ever	9 (0.4)	405 (0.5)	0.73 (0.37 - 1.44)
soc3d 23 20 Ever	-	Legal Support Workers - Ever	19 (0.9)	532 (0.7)	1.18 (0.74 - 1.89)
soc3d 23 20 Duration Category	1	Legal Support Workers - Duration (1)lt10yrs, (2)ge10yrs	8 (0.4)	267 (0.4)	1.02 (0.50 - 2.09)
soc3d 23 20 Duration Category	2	Legal Support Workers - Duration (1)lt10yrs, (2)ge10yrs	10 (0.5)	262 (0.4)	1.23 (0.65 - 2.35)
soc3d 25 10 Ever	-	Postsecondary Teachers - Ever	58 (2.8)	2454 (3.3)	0.88 (0.67 - 1.16)
soc3d 25 10 Duration Category	1	Postsecondary Teachers - Duration (1)lt10yrs, (2)ge10yrs	20 (1.0)	1025 (1.4)	0.72 (0.46 - 1.14)
soc3d 25 10 Duration Category	2	Postsecondary Teachers - Duration (1)lt10yrs, (2)ge10yrs	37 (1.8)	1405 (1.9)	0.98 (0.70 - 1.38)
soc3d 25 20 Ever	-	Preschool Primary Secondary and Special Education School Teachers - Ever	365 (17.6)	14823 (19.8)	1.06 (0.92 - 1.21)
soc3d 25 20 Duration Category	1	Preschool Primary Secondary and Special Education School Teachers - Duration (1)lt10yrs, (	121 (5.8)	5030 (6.7)	1.02 (0.83 - 1.24)
soc3d 25 20 Duration Category	2	Preschool Primary Secondary and Special Education School Teachers - Duration (1)lt10yrs, (	241 (11.6)	9672 (13.0)	1.08 (0.92 - 1.27)
soc3d 25 30 Ever	-	Other Teachers and Instructors - Ever	35 (1.7)	1492 (2.0)	0.91 (0.64 - 1.28)
soc3d 25 30 Duration Category	1	Other Teachers and Instructors - Duration (1)lt10yrs, (2)ge10yrs	25 (1.2)	749 (1.0)	1.28 (0.85 - 1.94)
soc3d 25 30 Duration Category	2	Other Teachers and Instructors - Duration (1)lt10yrs, (2)ge10yrs	10 (0.5)	727 (1.0)	0.54 (0.29 - 1.02)

soc3d 25 40 Ever	-	Librarians Curators and Archivists - Ever	52 (2.5)	1629 (2.2)	1.32 (0.99 - 1.76)
soc3d 25 40 Duration Category	1	Librarians Curators and Archivists - Duration (1)lt10yrs, (2)ge10yrs	21 (1.0)	599 (0.8)	1.35 (0.86 - 2.12)
soc3d 25 40 Duration Category	2	Librarians Curators and Archivists - Duration (1)lt10yrs, (2)ge10yrs	31 (1.5)	1019 (1.4)	1.32 (0.91 - 1.91)
soc3d 25 90 Ever	-	Other Education Training and Library Occupations - Ever	38 (1.8)	1816 (2.4)	0.92 (0.66 - 1.28)
soc3d 25 90 Duration Category	1	Other Education Training and Library Occupations - Duration (1)lt10yrs, (2)ge10yrs	21 (1.0)	1084 (1.5)	0.86 (0.56 - 1.34)
soc3d 25 90 Duration Category	2	Other Education Training and Library Occupations - Duration (1)lt10yrs, (2)ge10yrs	17 (0.8)	713 (1.0)	1.03 (0.63 - 1.68)
soc3d 27 10 Ever	-	Art and Design Workers - Ever	34 (1.6)	1304 (1.7)	0.86 (0.61 - 1.22)
soc3d 27 10 Duration Category	1	Art and Design Workers - Duration (1)lt10yrs, (2)ge10yrs	13 (0.6)	619 (0.8)	0.69 (0.39 - 1.21)
soc3d 27 10 Duration Category	2	Art and Design Workers - Duration (1)lt10yrs, (2)ge10yrs	21 (1.0)	651 (0.9)	1.07 (0.69 - 1.68)
soc3d 27 20 Ever	-	Entertainers and Performers Sports and Related Workers - Ever	26 (1.3)	822 (1.1)	1.11 (0.74 - 1.66)
soc3d 27 20 Duration Category	1	Entertainers and Performers Sports and Related Workers - Duration (1)lt10yrs, (2)ge10yrs	17 (0.8)	307 (0.4)	1.87 (1.12 - 3.10)
soc3d 27 20 Duration Category	2	Entertainers and Performers Sports and Related Workers - Duration (1)lt10yrs, (2)ge10yrs	9 (0.4)	483 (0.6)	0.68 (0.35 - 1.32)
soc3d 27 30 Ever	-	Media and Communication Workers - Ever	60 (2.9)	1796 (2.4)	1.01 (0.77 - 1.32)
soc3d 27 30 Duration Category	1	Media and Communication Workers - Duration (1)lt10yrs, (2)ge10yrs	35 (1.7)	1001 (1.3)	1.09 (0.77 - 1.55)
soc3d 27 30 Duration Category	2	Media and Communication Workers - Duration (1)lt10yrs, (2)ge10yrs	23 (1.1)	771 (1.0)	0.85 (0.55 - 1.30)
soc3d 27 40 Ever	-	Media and Communication Equipment Workers - Ever	5 (0.2)	157 (0.2)	1.01 (0.41 - 2.52)
soc3d 29 10 Ever	-	Health Diagnosing and Treating Practitioners - Ever	174 (8.4)	7098 (9.5)	0.87 (0.74 - 1.03)
soc3d 29 10 Duration Category	1	Health Diagnosing and Treating Practitioners - Duration (1)lt10yrs, (2)ge10yrs	46 (2.2)	2049 (2.7)	0.83 (0.61 - 1.12)
soc3d 29 10 Duration Category	2	Health Diagnosing and Treating Practitioners - Duration (1)lt10yrs, (2)ge10yrs	128 (6.2)	4993 (6.7)	0.90 (0.75 - 1.09)
soc3d 29 20 Ever	-	Health Technologists and Technicians - Ever	77 (3.7)	2724 (3.6)	1.06 (0.84 - 1.34)
soc3d 29 20 Duration Category	1	Health Technologists and Technicians - Duration (1)lt10yrs, (2)ge10yrs	35 (1.7)	1128 (1.5)	1.12 (0.79 - 1.58)
soc3d 29 20 Duration Category	2	Health Technologists and Technicians - Duration (1)lt10yrs, (2)ge10yrs	41 (2.0)	1570 (2.1)	1.01 (0.73 - 1.39)
soc3d 31 10 Ever	-	Nursing Psychiatric and Home Health Aides - Ever	34 (1.6)	1706 (2.3)	0.78 (0.55 - 1.11)
soc3d 31 10 Duration Category	1	Nursing Psychiatric and Home Health Aides - Duration (1)lt10yrs, (2)ge10yrs	18 (0.9)	1046 (1.4)	0.68 (0.42 - 1.10)
soc3d 31 10 Duration Category	2	Nursing Psychiatric and Home Health Aides - Duration (1)lt10yrs, (2)ge10yrs	14 (0.7)	631 (0.8)	0.84 (0.49 - 1.45)
soc3d 31 90 Ever	-	Other Healthcare Support Occupations - Ever	38 (1.8)	1419 (1.9)	1.00 (0.72 - 1.40)

soc3d 31 90 Duration Category	1	Other Healthcare Support Occupations - Duration (1)lt10yrs, (2)ge10yrs	22 (1.1)	845 (1.1)	0.97 (0.63 - 1.50)
soc3d 31 90 Duration Category	2	Other Healthcare Support Occupations - Duration (1)lt10yrs, (2)ge10yrs	16 (0.8)	558 (0.7)	1.08 (0.65 - 1.80)
soc3d 33 30 Ever	-	Law Enforcement Workers - Ever	8 (0.4)	175 (0.2)	1.30 (0.63 - 2.72)
soc3d 33 30 Duration Category	1	Law Enforcement Workers - Duration (1)lt10yrs, (2)ge10yrs	3 (0.1)	86 (0.1)	0.96 (0.29 - 3.13)
soc3d 33 30 Duration Category	2	Law Enforcement Workers - Duration (1)lt10yrs, (2)ge10yrs	5 (0.2)	87 (0.1)	1.68 (0.66 - 4.30)
soc3d 33 90 Ever	-	Other Protective Service Workers - Ever	9 (0.4)	386 (0.5)	0.77 (0.39 - 1.52)
soc3d 35 10 Ever	-	Supervisors of Food Preparation and Serving Workers - Ever	9 (0.4)	350 (0.5)	0.92 (0.47 - 1.80)
soc3d 35 10 Duration Category	1	Supervisors of Food Preparation and Serving Workers - Duration (1)lt10yrs, (2)ge10yrs	3 (0.1)	175 (0.2)	0.70 (0.22 - 2.23)
soc3d 35 10 Duration Category	2	Supervisors of Food Preparation and Serving Workers - Duration (1)lt10yrs, (2)ge10yrs	6 (0.3)	171 (0.2)	1.10 (0.48 - 2.53)
soc3d 35 20 Ever	-	Cooks and Food Preparation Workers - Ever	20 (1.0)	1142 (1.5)	0.73 (0.46 - 1.15)
soc3d 35 20 Duration Category	1	Cooks and Food Preparation Workers - Duration (1)lt10yrs, (2)ge10yrs	7 (0.3)	696 (0.9)	0.40 (0.19 - 0.85)
soc3d 35 20 Duration Category	2	Cooks and Food Preparation Workers - Duration (1)lt10yrs, (2)ge10yrs	13 (0.6)	416 (0.6)	1.38 (0.78 - 2.44)
soc3d 35 30 Ever	-	Food and Beverage Serving Workers - Ever	100 (4.8)	2514 (3.4)	1.23 (1.00 - 1.53)
soc3d 35 30 Duration Category	1	Food and Beverage Serving Workers - Duration (1)lt10yrs, (2)ge10yrs	70 (3.4)	1673 (2.2)	1.40 (1.09 - 1.81)
soc3d 35 30 Duration Category	2	Food and Beverage Serving Workers - Duration (1)lt10yrs, (2)ge10yrs	29 (1.4)	790 (1.1)	0.97 (0.66 - 1.43)
soc3d 35 90 Ever	-	Other Food Preparation and Serving Related Workers - Ever	18 (0.9)	497 (0.7)	1.42 (0.87 - 2.31)
soc3d 35 90 Duration Category	1	Other Food Preparation and Serving Related Workers - Duration (1)lt10yrs, (2)ge10yrs	13 (0.6)	356 (0.5)	1.40 (0.79 - 2.48)
soc3d 35 90 Duration Category	2	Other Food Preparation and Serving Related Workers - Duration (1)lt10yrs, (2)ge10yrs	4 (0.2)	132 (0.2)	1.21 (0.44 - 3.37)
soc3d 37 20 Ever	-	Building Cleaning and Pest Control Workers - Ever	26 (1.3)	1374 (1.8)	0.92 (0.61 - 1.38)
soc3d 37 20 Duration Category	1	Building Cleaning and Pest Control Workers - Duration (1)lt10yrs, (2)ge10yrs	16 (0.8)	739 (1.0)	1.02 (0.61 - 1.70)
soc3d 37 20 Duration Category	2	Building Cleaning and Pest Control Workers - Duration (1)lt10yrs, (2)ge10yrs	8 (0.4)	595 (0.8)	0.68 (0.33 - 1.38)
soc3d 39 20 Ever	-	Animal Care and Service Workers - Ever	4 (0.2)	70 (0.1)	1.68 (0.59 - 4.76)
soc3d 39 30 Ever	-	Entertainment Attendants and Related Workers - Ever	8 (0.4)	235 (0.3)	1.00 (0.48 - 2.06)
soc3d 39 30 Duration Category	1	Entertainment Attendants and Related Workers - Duration (1)lt10yrs, (2)ge10yrs	4 (0.2)	137 (0.2)	0.99 (0.36 - 2.74)
soc3d 39 30 Duration Category	2	Entertainment Attendants and Related Workers - Duration (1)lt10yrs, (2)ge10yrs	4 (0.2)	96 (0.1)	1.01 (0.36 - 2.85)
soc3d 39 50 Ever	-	Personal Appearance Workers - Ever	31 (1.5)	1013 (1.4)	1.10 (0.76 - 1.60)
soc3d 39 50 Duration Category	1	Personal Appearance Workers - Duration (1)lt10yrs, (2)ge10yrs	9 (0.4)	365 (0.5)	0.99 (0.50 - 1.94)

soc3d 39 50 Duration Category	2	Personal Appearance Workers - Duration (1)lt10yrs, (2)ge10yrs	21 (1.0)	626 (0.8)	1.13 (0.72 - 1.78)
soc3d 39 70 Ever	-	Tour and Travel Guides - Ever	6 (0.3)	137 (0.2)	1.88 (0.81 - 4.33)
soc3d 39 90 Ever	-	Other Personal Care and Service Workers - Ever	61 (2.9)	2144 (2.9)	1.27 (0.97 - 1.65)
soc3d 39 90 Duration Category	1	Other Personal Care and Service Workers - Duration (1)lt10yrs, (2)ge10yrs	45 (2.2)	1494 (2.0)	1.30 (0.96 - 1.77)
soc3d 39 90 Duration Category	2	Other Personal Care and Service Workers - Duration (1)lt10yrs, (2)ge10yrs	15 (0.7)	589 (0.8)	1.22 (0.72 - 2.07)
soc3d 41 10 Ever	-	Supervisors of Sales Workers - Ever	76 (3.7)	2178 (2.9)	1.10 (0.86 - 1.39)
soc3d 41 10 Duration Category	1	Supervisors of Sales Workers - Duration (1)lt10yrs, (2)ge10yrs	36 (1.7)	1047 (1.4)	1.06 (0.76 - 1.50)
soc3d 41 10 Duration Category	2	Supervisors of Sales Workers - Duration (1)lt10yrs, (2)ge10yrs	40 (1.9)	1109 (1.5)	1.16 (0.84 - 1.61)
soc3d 41 20 Ever	-	Retail Sales Workers - Ever	249 (12.0)	8888 (11.9)	1.02 (0.89 - 1.17)
soc3d 41 20 Duration Category	1	Retail Sales Workers - Duration (1)lt10yrs, (2)ge10yrs	175 (8.4)	6279 (8.4)	1.01 (0.86 - 1.18)
soc3d 41 20 Duration Category	2	Retail Sales Workers - Duration (1)lt10yrs, (2)ge10yrs	73 (3.5)	2458 (3.3)	1.09 (0.85 - 1.39)
soc3d 41 30 Ever	-	Sales Representatives Services - Ever	50 (2.4)	1827 (2.4)	0.83 (0.62 - 1.11)
soc3d 41 30 Duration Category	1	Sales Representatives Services - Duration (1)lt10yrs, (2)ge10yrs	25 (1.2)	992 (1.3)	0.82 (0.54 - 1.22)
soc3d 41 30 Duration Category	2	Sales Representatives Services - Duration (1)lt10yrs, (2)ge10yrs	25 (1.2)	817 (1.1)	0.87 (0.58 - 1.32)
soc3d 41 40 Ever	-	Sales Representatives Wholesale and Manufacturing - Ever	26 (1.3)	533 (0.7)	1.52 (1.01 - 2.29)
soc3d 41 40 Duration Category	1	Sales Representatives Wholesale and Manufacturing - Duration (1)lt10yrs, (2)ge10yrs	16 (0.8)	310 (0.4)	1.70 (1.01 - 2.86)
soc3d 41 40 Duration Category	2	Sales Representatives Wholesale and Manufacturing - Duration (1)lt10yrs, (2)ge10yrs	10 (0.5)	218 (0.3)	1.32 (0.69 - 2.55)
soc3d 41 90 Ever	-	Other Sales and Related Workers - Ever	101 (4.9)	3213 (4.3)	0.99 (0.80 - 1.21)
soc3d 41 90 Duration Category	1	Other Sales and Related Workers - Duration (1)lt10yrs, (2)ge10yrs	42 (2.0)	1636 (2.2)	0.81 (0.59 - 1.11)
soc3d 41 90 Duration Category	2	Other Sales and Related Workers - Duration (1)lt10yrs, (2)ge10yrs	57 (2.7)	1532 (2.1)	1.16 (0.88 - 1.53)
soc3d 43 10 Ever	-	Supervisors of Office and Administrative Support Workers - Ever	63 (3.0)	1833 (2.5)	1.06 (0.82 - 1.38)
soc3d 43 10 Duration Category	1	Supervisors of Office and Administrative Support Workers - Duration (1)lt10yrs, (2)ge10yrs	31 (1.5)	875 (1.2)	1.06 (0.73 - 1.53)
soc3d 43 10 Duration Category	2	Supervisors of Office and Administrative Support Workers - Duration (1)lt10yrs, (2)ge10yrs	32 (1.5)	935 (1.3)	1.09 (0.76 - 1.57)
soc3d 43 20 Ever	-	Communications Equipment Operators - Ever	41 (2.0)	1520 (2.0)	0.93 (0.68 - 1.29)
soc3d 43 20 Duration Category	1	Communications Equipment Operators - Duration (1)lt10yrs, (2)ge10yrs	32 (1.5)	1090 (1.5)	1.04 (0.72 - 1.49)
soc3d 43 20 Duration Category	2	Communications Equipment Operators - Duration (1)lt10yrs, (2)ge10yrs	9 (0.4)	416 (0.6)	0.70 (0.36 - 1.38)
soc3d 43 30 Ever	-	Financial Clerks - Ever	231 (11.1)	6943 (9.3)	1.17 (1.01 - 1.36)
soc3d 43 30 Duration Category	1	Financial Clerks - Duration (1)lt10yrs, (2)ge10yrs	146 (7.0)	3981 (5.3)	1.29 (1.08 - 1.54)

soc3d 43 30 Duration Category	2	Financial Clerks - Duration (1)lt10yrs, (2)ge10yrs	84 (4.1)	2907 (3.9)	1.02 (0.81 - 1.28)
soc3d 43 40 Ever	-	Information and Record Clerks - Ever	162 (7.8)	6333 (8.5)	0.88 (0.75 - 1.04)
soc3d 43 40 Duration Category	1	Information and Record Clerks - Duration (1)lt10yrs, (2)ge10yrs	105 (5.1)	4155 (5.6)	0.87 (0.71 - 1.07)
soc3d 43 40 Duration Category	2	Information and Record Clerks - Duration (1)lt10yrs, (2)ge10yrs	56 (2.7)	2091 (2.8)	0.93 (0.70 - 1.22)
soc3d 43 50 Ever	-	Material Recording Scheduling Dispatching and Distributing Workers - Ever	50 (2.4)	1768 (2.4)	0.99 (0.74 - 1.33)
soc3d 43 50 Duration Category	1	Material Recording Scheduling Dispatching and Distributing Workers - Duration (1)lt10yrs,	31 (1.5)	1036 (1.4)	1.03 (0.71 - 1.49)
soc3d 43 50 Duration Category	2	Material Recording Scheduling Dispatching and Distributing Workers - Duration (1)lt10yrs,	19 (0.9)	712 (1.0)	0.95 (0.60 - 1.53)
soc3d 43 60 Ever	-	Secretaries and Administrative Assistants - Ever	438 (21.1)	15993 (21.4)	0.93 (0.83 - 1.04)
soc3d 43 60 Duration Category	1	Secretaries and Administrative Assistants - Duration (1)lt10yrs, (2)ge10yrs	221 (10.7)	7859 (10.5)	0.97 (0.84 - 1.12)
soc3d 43 60 Duration Category	2	Secretaries and Administrative Assistants - Duration (1)lt10yrs, (2)ge10yrs	216 (10.4)	8016 (10.7)	0.90 (0.77 - 1.04)
soc3d 43 90 Ever	-	Other Office and Administrative Support Workers - Ever	320 (15.4)	11823 (15.8)	0.98 (0.87 - 1.12)
soc3d 43 90 Duration Category	1	Other Office and Administrative Support Workers - Duration (1)lt10yrs, (2)ge10yrs	206 (9.9)	7664 (10.3)	0.99 (0.85 - 1.15)
soc3d 43 90 Duration Category	2	Other Office and Administrative Support Workers - Duration (1)lt10yrs, (2)ge10yrs	110 (5.3)	4014 (5.4)	0.97 (0.79 - 1.18)
soc3d 45 20 Ever	-	Agricultural Workers - Ever	11 (0.5)	233 (0.3)	1.84 (0.98 - 3.46)
soc3d 47 20 Ever	-	Construction Trades Workers - Ever	12 (0.6)	235 (0.3)	1.63 (0.89 - 3.00)
soc3d 47 20 Duration Category	1	Construction Trades Workers - Duration (1)lt10yrs, (2)ge10yrs	7 (0.3)	132 (0.2)	1.69 (0.77 - 3.75)
soc3d 47 20 Duration Category	2	Construction Trades Workers - Duration (1)lt10yrs, (2)ge10yrs	5 (0.2)	98 (0.1)	1.62 (0.63 - 4.17)
soc3d 49 90 Ever	-	Other Installation Maintenance and Repair Occupations - Ever	4 (0.2)	106 (0.1)	1.48 (0.53 - 4.12)
soc3d 51 10 Ever	-	Supervisors of Production Workers - Ever	3 (0.1)	220 (0.3)	0.47 (0.15 - 1.47)
soc3d 51 20 Ever	-	Assemblers and Fabricators - Ever	29 (1.4)	786 (1.1)	1.31 (0.89 - 1.92)
soc3d 51 20 Duration Category	1	Assemblers and Fabricators - Duration (1)lt10yrs, (2)ge10yrs	22 (1.1)	471 (0.6)	1.65 (1.06 - 2.58)
soc3d 51 20 Duration Category	2	Assemblers and Fabricators - Duration (1)lt10yrs, (2)ge10yrs	7 (0.3)	305 (0.4)	0.80 (0.37 - 1.72)
soc3d 51 30 Ever	-	Food Processing Workers - Ever	5 (0.2)	272 (0.4)	0.86 (0.35 - 2.13)
soc3d 51 40 Ever	-	Metal Workers and Plastic Workers - Ever	17 (0.8)	437 (0.6)	1.37 (0.83 - 2.26)
soc3d 51 40 Duration Category	1	Metal Workers and Plastic Workers - Duration (1)lt10yrs, (2)ge10yrs	9 (0.4)	280 (0.4)	1.13 (0.57 - 2.23)
soc3d 51 40 Duration Category	2	Metal Workers and Plastic Workers - Duration (1)lt10yrs, (2)ge10yrs	8 (0.4)	150 (0.2)	1.85 (0.88 - 3.91)
soc3d 51 51 Ever	-	Printing Workers - Ever	13 (0.6)	292 (0.4)	1.39 (0.78 - 2.47)
soc3d 51 51 Duration Category	1	Printing Workers - Duration (1)lt10yrs, (2)ge10yrs	8 (0.4)	149 (0.2)	1.62 (0.78 - 3.40)
soc3d 51 51 Duration Category	2	Printing Workers - Duration (1)lt10yrs, (2)ge10yrs	5 (0.2)	136 (0.2)	1.17 (0.47 - 2.94)

soc3d 51 60 Ever	-	Textile Apparel and Furnishings Workers - Ever	34 (1.6)	1480 (2.0)	1.00 (0.70 - 1.43)
soc3d 51 60 Duration Category	1	Textile Apparel and Furnishings Workers - Duration (1)lt10yrs, (2)ge10yrs	13 (0.6)	787 (1.1)	0.74 (0.42 - 1.30)
soc3d 51 60 Duration Category	2	Textile Apparel and Furnishings Workers - Duration (1)lt10yrs, (2)ge10yrs	20 (1.0)	666 (0.9)	1.25 (0.79 - 1.99)
soc3d 51 90 Ever	-	Other Production Occupations - Ever	53 (2.6)	2107 (2.8)	0.94 (0.71 - 1.25)
soc3d 51 90 Duration Category	1	Other Production Occupations - Duration (1)lt10yrs, (2)ge10yrs	31 (1.5)	1336 (1.8)	0.84 (0.58 - 1.21)
soc3d 51 90 Duration Category	2	Other Production Occupations - Duration (1)lt10yrs, (2)ge10yrs	21 (1.0)	729 (1.0)	1.15 (0.73 - 1.79)
soc3d 53 20 Ever	-	Air Transportation Workers - Ever	12 (0.6)	347 (0.5)	1.10 (0.61 - 1.99)
soc3d 53 30 Ever	-	Motor Vehicle Operators - Ever	18 (0.9)	576 (0.8)	1.01 (0.62 - 1.63)
soc3d 53 30 Duration Category	1	Motor Vehicle Operators - Duration (1)lt10yrs, (2)ge10yrs	10 (0.5)	312 (0.4)	0.97 (0.51 - 1.85)
soc3d 53 30 Duration Category	2	Motor Vehicle Operators - Duration (1)lt10yrs, (2)ge10yrs	7 (0.3)	256 (0.3)	0.95 (0.44 - 2.05)
soc3d 53 70 Ever	-	Material Moving Workers - Ever	15 (0.7)	571 (0.8)	1.02 (0.60 - 1.73)
soc3d 53 70 Duration Category	1	Material Moving Workers - Duration (1)lt10yrs, (2)ge10yrs	10 (0.5)	400 (0.5)	1.00 (0.52 - 1.90)
soc3d 53 70 Duration Category	2	Material Moving Workers - Duration (1)lt10yrs, (2)ge10yrs	5 (0.2)	159 (0.2)	1.14 (0.46 - 2.85)
soc5d 11 101 Ever	-	Chief Executives - Ever	25 (1.2)	532 (0.7)	1.41 (0.93 - 2.14)
soc5d 11 101 Duration Category	1	Chief Executives - Duration (1)lt10yrs, (2)ge10yrs	9 (0.4)	210 (0.3)	1.33 (0.67 - 2.65)
soc5d 11 101 Duration Category	2	Chief Executives - Duration (1)lt10yrs, (2)ge10yrs	15 (0.7)	317 (0.4)	1.37 (0.80 - 2.35)
soc5d 11 102 Ever	-	General and Operations Managers - Ever	17 (0.8)	637 (0.9)	0.90 (0.55 - 1.47)
soc5d 11 102 Duration Category	1	General and Operations Managers - Duration (1)lt10yrs, (2)ge10yrs	8 (0.4)	366 (0.5)	0.77 (0.38 - 1.57)
soc5d 11 102 Duration Category	2	General and Operations Managers - Duration (1)lt10yrs, (2)ge10yrs	8 (0.4)	268 (0.4)	0.94 (0.46 - 1.92)
soc5d 11 201 Ever	-	Advertising and Promotions Managers - Ever	9 (0.4)	91 (0.1)	2.58 (1.26 - 5.27)
soc5d 11 202 Ever	-	Marketing and Sales Managers - Ever	8 (0.4)	402 (0.5)	0.67 (0.33 - 1.36)
soc5d 11 202 Duration Category	1	Marketing and Sales Managers - Duration (1)lt10yrs, (2)ge10yrs	3 (0.1)	247 (0.3)	0.44 (0.14 - 1.40)
soc5d 11 202 Duration Category	2	Marketing and Sales Managers - Duration (1)lt10yrs, (2)ge10yrs	5 (0.2)	153 (0.2)	0.98 (0.39 - 2.43)
soc5d 11 203 Ever	-	Public Relations and Fundraising Managers - Ever	4 (0.2)	120 (0.2)	1.04 (0.38 - 2.88)
soc5d 11 302 Ever	-	Computer and Information Systems Managers - Ever	3 (0.1)	57 (0.1)	1.39 (0.42 - 4.64)
soc5d 11 303 Ever	-	Financial Managers - Ever	23 (1.1)	692 (0.9)	1.09 (0.71 - 1.67)
soc5d 11 303 Duration Category	1	Financial Managers - Duration (1)lt10yrs, (2)ge10yrs	12 (0.6)	268 (0.4)	1.57 (0.87 - 2.85)
soc5d 11 303 Duration Category	2	Financial Managers - Duration (1)lt10yrs, (2)ge10yrs	11 (0.5)	420 (0.6)	0.82 (0.44 - 1.51)
soc5d 11 306 Ever	-	Purchasing Managers - Ever	4 (0.2)	88 (0.1)	1.30 (0.46 - 3.64)
soc5d 11 307 Ever	-	Transportation Storage and Distribution Managers - Ever	4 (0.2)	48 (0.1)	2.97 (1.03 - 8.59)

soc5d 11 312 Ever	-	Human Resources Managers - Ever	10 (0.5)	267 (0.4)	1.30 (0.68 - 2.49)
soc5d 11 312 Duration Category	1	Human Resources Managers - Duration (1)lt10yrs, (2)ge10yrs	3 (0.1)	130 (0.2)	0.75 (0.23 - 2.40)
soc5d 11 312 Duration Category	2	Human Resources Managers - Duration (1)lt10yrs, (2)ge10yrs	7 (0.3)	135 (0.2)	1.90 (0.87 - 4.16)
soc5d 11 313 Ever	-	Training and Development Managers - Ever	4 (0.2)	59 (0.1)	2.48 (0.86 - 7.09)
soc5d 11 901 Ever	-	Farmers Ranchers and Other Agricultural Managers - Ever	4 (0.2)	213 (0.3)	0.70 (0.25 - 1.91)
soc5d 11 903 Ever	-	Education Administrators - Ever	40 (1.9)	1755 (2.3)	0.87 (0.63 - 1.20)
soc5d 11 903 Duration Category	1	Education Administrators - Duration (1)lt10yrs, (2)ge10yrs	15 (0.7)	726 (1.0)	0.80 (0.47 - 1.35)
soc5d 11 903 Duration Category	2	Education Administrators - Duration (1)lt10yrs, (2)ge10yrs	25 (1.2)	1023 (1.4)	0.92 (0.61 - 1.39)
soc5d 11 905 Ever	-	Food Service Managers - Ever	14 (0.7)	473 (0.6)	0.85 (0.49 - 1.47)
soc5d 11 905 Duration Category	1	Food Service Managers - Duration (1)lt10yrs, (2)ge10yrs	6 (0.3)	239 (0.3)	0.66 (0.29 - 1.52)
soc5d 11 905 Duration Category	2	Food Service Managers - Duration (1)lt10yrs, (2)ge10yrs	8 (0.4)	228 (0.3)	1.09 (0.53 - 2.24)
soc5d 11 908 Ever	-	Lodging Managers - Ever	4 (0.2)	214 (0.3)	0.56 (0.20 - 1.52)
soc5d 11 911 Ever	-	Medical and Health Services Managers - Ever	27 (1.3)	841 (1.1)	1.08 (0.73 - 1.61)
soc5d 11 911 Duration Category	1	Medical and Health Services Managers - Duration (1)lt10yrs, (2)ge10yrs	11 (0.5)	408 (0.5)	0.97 (0.53 - 1.80)
soc5d 11 911 Duration Category	2	Medical and Health Services Managers - Duration (1)lt10yrs, (2)ge10yrs	16 (0.8)	427 (0.6)	1.19 (0.72 - 1.99)
soc5d 11 914 Ever	-	Property Real Estate and Community Association Managers - Ever	18 (0.9)	537 (0.7)	1.00 (0.62 - 1.62)
soc5d 11 914 Duration Category	1	Property Real Estate and Community Association Managers - Duration (1)lt10yrs, (2)ge10yrs	9 (0.4)	250 (0.3)	0.97 (0.49 - 1.92)
soc5d 11 914 Duration Category	2	Property Real Estate and Community Association Managers - Duration (1)lt10yrs, (2)ge10yrs	9 (0.4)	278 (0.4)	1.08 (0.54 - 2.12)
soc5d 11 915 Ever	-	Social and Community Service Managers - Ever	24 (1.2)	645 (0.9)	1.36 (0.89 - 2.07)
soc5d 11 915 Duration Category	1	Social and Community Service Managers - Duration (1)lt10yrs, (2)ge10yrs	20 (1.0)	381 (0.5)	2.06 (1.29 - 3.29)
soc5d 11 915 Duration Category	2	Social and Community Service Managers - Duration (1)lt10yrs, (2)ge10yrs	3 (0.1)	253 (0.3)	0.38 (0.12 - 1.21)
soc5d 11 919 Ever	-	Miscellaneous Managers - Ever	107 (5.2)	2929 (3.9)	1.18 (0.96 - 1.45)
soc5d 11 919 Duration Category	1	Miscellaneous Managers - Duration (1)lt10yrs, (2)ge10yrs	53 (2.6)	1321 (1.8)	1.35 (1.02 - 1.80)
soc5d 11 919 Duration Category	2	Miscellaneous Managers - Duration (1)lt10yrs, (2)ge10yrs	53 (2.6)	1577 (2.1)	1.05 (0.79 - 1.39)
soc5d 13 102 Ever	-	Buyers and Purchasing Agents - Ever	30 (1.4)	730 (1.0)	1.27 (0.87 - 1.85)
soc5d 13 102 Duration Category	1	Buyers and Purchasing Agents - Duration (1)lt10yrs, (2)ge10yrs	15 (0.7)	416 (0.6)	1.03 (0.61 - 1.75)
soc5d 13 102 Duration Category	2	Buyers and Purchasing Agents - Duration (1)lt10yrs, (2)ge10yrs	15 (0.7)	310 (0.4)	1.65 (0.97 - 2.83)
soc5d 13 103 Ever	-	Claims Adjusters Appraisers Examiners and Investigators - Ever	5 (0.2)	251 (0.3)	0.64 (0.26 - 1.58)
soc5d 13 104 Ever	-	Compliance Officers - Ever	3 (0.1)	105 (0.1)	0.98 (0.31 - 3.17)
soc5d 13 107 Ever	-	Human Resources Workers - Ever	17 (0.8)	622 (0.8)	0.82 (0.50 - 1.35)

soc5d 13 107 Duration Category	1	Human Resources Workers - Duration (1)lt10yrs, (2)ge10yrs	12 (0.6)	367 (0.5)	0.95 (0.52 - 1.71)
soc5d 13 107 Duration Category	2	Human Resources Workers - Duration (1)lt10yrs, (2)ge10yrs	5 (0.2)	244 (0.3)	0.66 (0.27 - 1.61)
soc5d 13 111 Ever	-	Management Analysts - Ever	10 (0.5)	331 (0.4)	1.03 (0.54 - 1.96)
soc5d 13 111 Duration Category	1	Management Analysts - Duration (1)lt10yrs, (2)ge10yrs	7 (0.3)	186 (0.2)	1.29 (0.60 - 2.79)
soc5d 13 111 Duration Category	2	Management Analysts - Duration (1)lt10yrs, (2)ge10yrs	3 (0.1)	139 (0.2)	0.72 (0.23 - 2.31)
soc5d 13 116 Ever	-	Market Research Analysts and Marketing Specialists - Ever	8 (0.4)	178 (0.2)	1.64 (0.80 - 3.38)
soc5d 13 116 Duration Category	1	Market Research Analysts and Marketing Specialists - Duration (1)lt10yrs, (2)ge10yrs	4 (0.2)	115 (0.2)	1.26 (0.46 - 3.47)
soc5d 13 116 Duration Category	2	Market Research Analysts and Marketing Specialists - Duration (1)lt10yrs, (2)ge10yrs	4 (0.2)	60 (0.1)	2.50 (0.88 - 7.13)
soc5d 13 119 Ever	-	Miscellaneous Business Operations Specialists - Ever	19 (0.9)	597 (0.8)	1.05 (0.66 - 1.68)
soc5d 13 201 Ever	-	Accountants and Auditors - Ever	52 (2.5)	1503 (2.0)	1.12 (0.84 - 1.49)
soc5d 13 201 Duration Category	1	Accountants and Auditors - Duration (1)lt10yrs, (2)ge10yrs	16 (0.8)	668 (0.9)	0.81 (0.49 - 1.35)
soc5d 13 201 Duration Category	2	Accountants and Auditors - Duration (1)lt10yrs, (2)ge10yrs	36 (1.7)	830 (1.1)	1.35 (0.95 - 1.91)
soc5d 13 203 Ever	-	Budget Analysts - Ever	3 (0.1)	97 (0.1)	0.98 (0.30 - 3.17)
soc5d 13 205 Ever	-	Financial Analysts and Advisors - Ever	15 (0.7)	271 (0.4)	1.94 (1.13 - 3.33)
soc5d 13 205 Duration Category	1	Financial Analysts and Advisors - Duration (1)lt10yrs, (2)ge10yrs	8 (0.4)	110 (0.1)	2.86 (1.36 - 6.03)
soc5d 13 205 Duration Category	2	Financial Analysts and Advisors - Duration (1)lt10yrs, (2)ge10yrs	6 (0.3)	160 (0.2)	1.21 (0.52 - 2.79)
soc5d 13 207 Ever	-	Credit Counselors and Loan Officers - Ever	12 (0.6)	201 (0.3)	1.92 (1.05 - 3.52)
soc5d 13 207 Duration Category	1	Credit Counselors and Loan Officers - Duration (1)lt10yrs, (2)ge10yrs	7 (0.3)	85 (0.1)	2.54 (1.13 - 5.70)
soc5d 13 207 Duration Category	2	Credit Counselors and Loan Officers - Duration (1)lt10yrs, (2)ge10yrs	5 (0.2)	114 (0.2)	1.45 (0.58 - 3.65)
soc5d 13 208 Ever	-	Tax Examiners Collectors and Preparers and Revenue Agents - Ever	17 (0.8)	451 (0.6)	1.20 (0.73 - 1.98)
soc5d 13 208 Duration Category	1	Tax Examiners Collectors and Preparers and Revenue Agents - Duration (1)lt10yrs, (2)ge10yr	8 (0.4)	232 (0.3)	1.12 (0.54 - 2.30)
soc5d 13 208 Duration Category	2	Tax Examiners Collectors and Preparers and Revenue Agents - Duration (1)lt10yrs, (2)ge10yr	9 (0.4)	213 (0.3)	1.31 (0.66 - 2.62)
soc5d 15 112 Ever	-	Computer and Information Analysts - Ever	9 (0.4)	337 (0.5)	0.83 (0.42 - 1.63)
soc5d 15 112 Duration Category	1	Computer and Information Analysts - Duration (1)lt10yrs, (2)ge10yrs	4 (0.2)	148 (0.2)	0.86 (0.31 - 2.36)
soc5d 15 112 Duration Category	2	Computer and Information Analysts - Duration (1)lt10yrs, (2)ge10yrs	5 (0.2)	189 (0.3)	0.81 (0.33 - 1.99)
soc5d 15 113 Ever	-	Software Developers and Programmers - Ever	16 (0.8)	520 (0.7)	1.17 (0.70 - 1.95)
soc5d 15 113 Duration Category	1	Software Developers and Programmers - Duration (1)lt10yrs, (2)ge10yrs	6 (0.3)	236 (0.3)	1.03 (0.45 - 2.35)
soc5d 15 113 Duration Category	2	Software Developers and Programmers - Duration (1)lt10yrs, (2)ge10yrs	10 (0.5)	282 (0.4)	1.28 (0.67 - 2.45)

soc5d 15 114 Ever	-	Database and Systems Administrators and Network Architects - Ever	3 (0.1)	77 (0.1)	1.08 (0.33 - 3.57)
soc5d 15 202 Ever	-	Mathematicians - Ever	3 (0.1)	43 (0.1)	2.64 (0.78 - 8.90)
soc5d 15 203 Ever	-	Operations Research Analysts - Ever	10 (0.5)	262 (0.4)	1.32 (0.69 - 2.52)
soc5d 15 203 Duration Category	1	Operations Research Analysts - Duration (1)lt10yrs, (2)ge10yrs	5 (0.2)	121 (0.2)	1.48 (0.59 - 3.69)
soc5d 15 203 Duration Category	2	Operations Research Analysts - Duration (1)lt10yrs, (2)ge10yrs	5 (0.2)	139 (0.2)	1.19 (0.48 - 2.97)
soc5d 15 204 Ever	-	Statisticians - Ever	7 (0.3)	127 (0.2)	1.82 (0.83 - 3.99)
soc5d 17 201 Ever	-	Aerospace Engineers - Ever	3 (0.1)	28 (0.0)	3.09 (0.88 - 10.85)
soc5d 17 301 Ever	-	Drafters - Ever	5 (0.2)	201 (0.3)	0.70 (0.28 - 1.73)
soc5d 17 302 Ever	-	Engineering Technicians Except Drafters - Ever	14 (0.7)	309 (0.4)	1.49 (0.86 - 2.60)
soc5d 19 102 Ever	-	Biological Scientists - Ever	10 (0.5)	211 (0.3)	1.81 (0.94 - 3.49)
soc5d 19 102 Duration Category	1	Biological Scientists - Duration (1)lt10yrs, (2)ge10yrs	3 (0.1)	104 (0.1)	1.11 (0.35 - 3.59)
soc5d 19 102 Duration Category	2	Biological Scientists - Duration (1)lt10yrs, (2)ge10yrs	7 (0.3)	106 (0.1)	2.62 (1.18 - 5.84)
soc5d 19 104 Ever	-	Medical Scientists - Ever	4 (0.2)	124 (0.2)	1.48 (0.54 - 4.08)
soc5d 19 203 Ever	-	Chemists and Materials Scientists - Ever	10 (0.5)	264 (0.4)	1.37 (0.71 - 2.65)
soc5d 19 203 Duration Category	1	Chemists and Materials Scientists - Duration (1)lt10yrs, (2)ge10yrs	6 (0.3)	178 (0.2)	1.14 (0.49 - 2.64)
soc5d 19 203 Duration Category	2	Chemists and Materials Scientists - Duration (1)lt10yrs, (2)ge10yrs	3 (0.1)	84 (0.1)	1.58 (0.49 - 5.14)
soc5d 19 209 Ever	-	Miscellaneous Physical Scientists - Ever	4 (0.2)	170 (0.2)	1.14 (0.42 - 3.14)
soc5d 19 301 Ever	-	Economists - Ever	3 (0.1)	67 (0.1)	1.57 (0.48 - 5.17)
soc5d 19 303 Ever	-	Psychologists - Ever	21 (1.0)	555 (0.7)	1.22 (0.78 - 1.92)
soc5d 19 303 Duration Category	1	Psychologists - Duration (1)lt10yrs, (2)ge10yrs	6 (0.3)	131 (0.2)	1.40 (0.60 - 3.26)
soc5d 19 303 Duration Category	2	Psychologists - Duration (1)lt10yrs, (2)ge10yrs	15 (0.7)	420 (0.6)	1.17 (0.69 - 1.99)
soc5d 19 403 Ever	-	Chemical Technicians - Ever	3 (0.1)	144 (0.2)	0.80 (0.25 - 2.57)
soc5d 19 409 Ever	-	Miscellaneous Life Physical and Social Science Technicians - Ever	17 (0.8)	622 (0.8)	1.02 (0.62 - 1.68)
soc5d 19 409 Duration Category	1	Miscellaneous Life Physical and Social Science Technicians - Duration (1)lt10yrs, (2)ge10y	11 (0.5)	439 (0.6)	0.94 (0.51 - 1.74)
soc5d 19 409 Duration Category	2	Miscellaneous Life Physical and Social Science Technicians - Duration (1)lt10yrs, (2)ge10y	6 (0.3)	182 (0.2)	1.22 (0.53 - 2.81)
soc5d 21 101 Ever	-	Counselors - Ever	39 (1.9)	1307 (1.7)	1.09 (0.78 - 1.51)
soc5d 21 101 Duration Category	1	Counselors - Duration (1)lt10yrs, (2)ge10yrs	13 (0.6)	666 (0.9)	0.71 (0.41 - 1.24)
soc5d 21 101 Duration Category	2	Counselors - Duration (1)lt10yrs, (2)ge10yrs	25 (1.2)	632 (0.8)	1.44 (0.95 - 2.19)
soc5d 21 102 Ever	-	Social Workers - Ever	49 (2.4)	2071 (2.8)	0.79 (0.59 - 1.06)
soc5d 21 102 Duration Category	1	Social Workers - Duration (1)lt10yrs, (2)ge10yrs	26 (1.3)	1003 (1.3)	0.91 (0.61 - 1.36)

soc5d 21 102 Duration Category	2	Social Workers - Duration (1)lt10yrs, (2)ge10yrs	22 (1.1)	1043 (1.4)	0.67 (0.43 - 1.03)
soc5d 21 109 Ever	-	Miscellaneous Community and Social Service Specialists - Ever	19 (0.9)	545 (0.7)	1.23 (0.77 - 1.97)
soc5d 21 109 Duration Category	1	Miscellaneous Community and Social Service Specialists - Duration (1)lt10yrs, (2)ge10yrs	11 (0.5)	330 (0.4)	1.24 (0.67 - 2.30)
soc5d 21 109 Duration Category	2	Miscellaneous Community and Social Service Specialists - Duration (1)lt10yrs, (2)ge10yrs	8 (0.4)	206 (0.3)	1.23 (0.60 - 2.55)
soc5d 21 201 Ever	-	Clergy - Ever	4 (0.2)	159 (0.2)	1.36 (0.50 - 3.72)
soc5d 21 209 Ever	-	Miscellaneous Religious Workers - Ever	3 (0.1)	225 (0.3)	0.68 (0.21 - 2.16)
soc5d 23 101 Ever	-	Lawyers and Judicial Law Clerks - Ever	8 (0.4)	369 (0.5)	0.73 (0.36 - 1.50)
soc5d 23 201 Ever	-	Paralegals and Legal Assistants - Ever	14 (0.7)	289 (0.4)	1.53 (0.88 - 2.66)
soc5d 23 201 Duration Category	1	Paralegals and Legal Assistants - Duration (1)lt10yrs, (2)ge10yrs	6 (0.3)	140 (0.2)	1.40 (0.60 - 3.26)
soc5d 23 201 Duration Category	2	Paralegals and Legal Assistants - Duration (1)lt10yrs, (2)ge10yrs	7 (0.3)	147 (0.2)	1.45 (0.67 - 3.16)
soc5d 23 209 Ever	-	Miscellaneous Legal Support Workers - Ever	5 (0.2)	252 (0.3)	0.68 (0.28 - 1.67)
soc5d 25 201 Ever	-	Preschool and Kindergarten Teachers - Ever	20 (1.0)	1058 (1.4)	0.83 (0.53 - 1.31)
soc5d 25 201 Duration Category	1	Preschool and Kindergarten Teachers - Duration (1)lt10yrs, (2)ge10yrs	9 (0.4)	569 (0.8)	0.71 (0.36 - 1.38)
soc5d 25 201 Duration Category	2	Preschool and Kindergarten Teachers - Duration (1)lt10yrs, (2)ge10yrs	11 (0.5)	482 (0.6)	0.98 (0.53 - 1.80)
soc5d 25 202 Ever	-	Elementary and Middle School Teachers - Ever	293 (14.1)	12137 (16.2)	1.03 (0.89 - 1.19)
soc5d 25 202 Duration Category	1	Elementary and Middle School Teachers - Duration (1)lt10yrs, (2)ge10yrs	88 (4.2)	3943 (5.3)	0.94 (0.75 - 1.18)
soc5d 25 202 Duration Category	2	Elementary and Middle School Teachers - Duration (1)lt10yrs, (2)ge10yrs	202 (9.7)	8088 (10.8)	1.08 (0.91 - 1.28)
soc5d 25 203 Ever	-	Secondary School Teachers - Ever	50 (2.4)	2005 (2.7)	1.02 (0.76 - 1.38)
soc5d 25 203 Duration Category	1	Secondary School Teachers - Duration (1)lt10yrs, (2)ge10yrs	28 (1.3)	1001 (1.3)	1.18 (0.80 - 1.74)
soc5d 25 203 Duration Category	2	Secondary School Teachers - Duration (1)lt10yrs, (2)ge10yrs	22 (1.1)	985 (1.3)	0.89 (0.57 - 1.38)
soc5d 25 205 Ever	-	Special Education Teachers - Ever	7 (0.3)	229 (0.3)	1.27 (0.59 - 2.74)
soc5d 25 401 Ever	-	Archivists Curators and Museum Technicians - Ever	8 (0.4)	136 (0.2)	2.55 (1.22 - 5.32)
soc5d 25 401 Duration Category	1	Archivists Curators and Museum Technicians - Duration (1)lt10yrs, (2)ge10yrs	4 (0.2)	73 (0.1)	2.29 (0.81 - 6.47)
soc5d 25 401 Duration Category	2	Archivists Curators and Museum Technicians - Duration (1)lt10yrs, (2)ge10yrs	4 (0.2)	63 (0.1)	2.88 (1.02 - 8.11)
soc5d 25 402 Ever	-	Librarians - Ever	41 (2.0)	1408 (1.9)	1.20 (0.87 - 1.65)
soc5d 25 402 Duration Category	1	Librarians - Duration (1)lt10yrs, (2)ge10yrs	15 (0.7)	480 (0.6)	1.17 (0.69 - 1.99)
soc5d 25 402 Duration Category	2	Librarians - Duration (1)lt10yrs, (2)ge10yrs	26 (1.3)	917 (1.2)	1.23 (0.82 - 1.84)
soc5d 25 403 Ever	-	Library Technicians - Ever	3 (0.1)	111 (0.1)	1.03 (0.32 - 3.31)
soc5d 25 901 Ever	-	AudioVisual and Multimedia Collections Specialists - Ever	8 (0.4)	428 (0.6)	0.74 (0.36 - 1.51)

soc5d 25 901 Duration Category	1	AudioVisual and Multimedia Collections Specialists - Duration (1)lt10yrs, (2)ge10yrs	3 (0.1)	268 (0.4)	0.45 (0.14 - 1.41)
soc5d 25 901 Duration Category	2	AudioVisual and Multimedia Collections Specialists - Duration (1)lt10yrs, (2)ge10yrs	5 (0.2)	158 (0.2)	1.25 (0.50 - 3.10)
soc5d 25 902 Ever	-	Farm and Home Management Advisors - Ever	8 (0.4)	428 (0.6)	0.74 (0.36 - 1.51)
soc5d 25 902 Duration Category	1	Farm and Home Management Advisors - Duration (1)lt10yrs, (2)ge10yrs	3 (0.1)	268 (0.4)	0.45 (0.14 - 1.41)
soc5d 25 902 Duration Category	2	Farm and Home Management Advisors - Duration (1)lt10yrs, (2)ge10yrs	5 (0.2)	158 (0.2)	1.25 (0.50 - 3.10)
soc5d 25 903 Ever	-	Instructional Coordinators - Ever	8 (0.4)	428 (0.6)	0.74 (0.36 - 1.51)
soc5d 25 903 Duration Category	1	Instructional Coordinators - Duration (1)lt10yrs, (2)ge10yrs	3 (0.1)	268 (0.4)	0.45 (0.14 - 1.41)
soc5d 25 903 Duration Category	2	Instructional Coordinators - Duration (1)lt10yrs, (2)ge10yrs	5 (0.2)	158 (0.2)	1.25 (0.50 - 3.10)
soc5d 25 904 Ever	-	Teacher Assistants - Ever	30 (1.4)	1390 (1.9)	0.99 (0.68 - 1.43)
soc5d 25 904 Duration Category	1	Teacher Assistants - Duration (1)lt10yrs, (2)ge10yrs	18 (0.9)	817 (1.1)	1.02 (0.63 - 1.65)
soc5d 25 904 Duration Category	2	Teacher Assistants - Duration (1)lt10yrs, (2)ge10yrs	12 (0.6)	556 (0.7)	0.95 (0.53 - 1.71)
soc5d 25 909 Ever	-	Miscellaneous Education Training and Library Workers - Ever	8 (0.4)	428 (0.6)	0.74 (0.36 - 1.51)
soc5d 25 909 Duration Category	1	Miscellaneous Education Training and Library Workers - Duration (1)lt10yrs, (2)ge10yrs	3 (0.1)	268 (0.4)	0.45 (0.14 - 1.41)
soc5d 25 909 Duration Category	2	Miscellaneous Education Training and Library Workers - Duration (1)lt10yrs, (2)ge10yrs	5 (0.2)	158 (0.2)	1.25 (0.50 - 3.10)
soc5d 27 101 Ever	-	Artists and Related Workers - Ever	11 (0.5)	452 (0.6)	0.77 (0.42 - 1.41)
soc5d 27 101 Duration Category	1	Artists and Related Workers - Duration (1)lt10yrs, (2)ge10yrs	5 (0.2)	192 (0.3)	0.82 (0.33 - 2.03)
soc5d 27 101 Duration Category	2	Artists and Related Workers - Duration (1)lt10yrs, (2)ge10yrs	6 (0.3)	241 (0.3)	0.80 (0.35 - 1.82)
soc5d 27 102 Ever	-	Designers - Ever	23 (1.1)	889 (1.2)	0.87 (0.57 - 1.33)
soc5d 27 102 Duration Category	1	Designers - Duration (1)lt10yrs, (2)ge10yrs	8 (0.4)	454 (0.6)	0.58 (0.29 - 1.19)
soc5d 27 102 Duration Category	2	Designers - Duration (1)lt10yrs, (2)ge10yrs	15 (0.7)	419 (0.6)	1.21 (0.71 - 2.05)
soc5d 27 201 Ever	-	Actors Producers and Directors - Ever	5 (0.2)	214 (0.3)	0.61 (0.25 - 1.50)
soc5d 27 202 Ever	-	Athletes Coaches Umpires and Related Workers - Ever	7 (0.3)	162 (0.2)	1.48 (0.68 - 3.23)
soc5d 27 203 Ever	-	Dancers and Choreographers - Ever	4 (0.2)	59 (0.1)	2.35 (0.82 - 6.70)
soc5d 27 204 Ever	-	Musicians Singers and Related Workers - Ever	10 (0.5)	357 (0.5)	1.25 (0.65 - 2.38)
soc5d 27 204 Duration Category	1	Musicians Singers and Related Workers - Duration (1)lt10yrs, (2)ge10yrs	4 (0.2)	80 (0.1)	1.73 (0.60 - 4.94)
soc5d 27 204 Duration Category	2	Musicians Singers and Related Workers - Duration (1)lt10yrs, (2)ge10yrs	6 (0.3)	260 (0.3)	1.14 (0.50 - 2.60)
soc5d 27 302 Ever	-	News Analysts Reporters and Correspondents - Ever	9 (0.4)	363 (0.5)	0.62 (0.32 - 1.23)
soc5d 27 303 Ever	-	Public Relations Specialists - Ever	16 (0.8)	307 (0.4)	1.60 (0.95 - 2.69)
soc5d 27 303 Duration Category	1	Public Relations Specialists - Duration (1)lt10yrs, (2)ge10yrs	11 (0.5)	204 (0.3)	1.64 (0.88 - 3.06)

soc5d 27 303 Duration Category	2	Public Relations Specialists - Duration (1)lt10yrs, (2)ge10yrs	4 (0.2)	100 (0.1)	1.21 (0.43 - 3.38)
soc5d 27 304 Ever	-	Writers and Editors - Ever	41 (2.0)	1117 (1.5)	1.12 (0.81 - 1.54)
soc5d 27 304 Duration Category	1	Writers and Editors - Duration (1)lt10yrs, (2)ge10yrs	28 (1.4)	627 (0.8)	1.40 (0.95 - 2.08)
soc5d 27 304 Duration Category	2	Writers and Editors - Duration (1)lt10yrs, (2)ge10yrs	12 (0.6)	474 (0.6)	0.73 (0.40 - 1.30)
soc5d 27 402 Ever	-	Photographers - Ever	5 (0.2)	107 (0.1)	1.52 (0.60 - 3.83)
soc5d 29 103 Ever	-	Dietitians and Nutritionists - Ever	6 (0.3)	414 (0.6)	0.56 (0.25 - 1.27)
soc5d 29 103 Duration Category	1	Dietitians and Nutritionists - Duration (1)lt10yrs, (2)ge10yrs	3 (0.1)	214 (0.3)	0.60 (0.19 - 1.91)
soc5d 29 103 Duration Category	2	Dietitians and Nutritionists - Duration (1)lt10yrs, (2)ge10yrs	3 (0.1)	195 (0.3)	0.55 (0.17 - 1.73)
soc5d 29 105 Ever	-	Pharmacists - Ever	4 (0.2)	109 (0.1)	1.52 (0.54 - 4.28)
soc5d 29 106 Ever	-	Physicians and Surgeons - Ever	6 (0.3)	228 (0.3)	1.08 (0.47 - 2.48)
soc5d 29 106 Duration Category	1	Physicians and Surgeons - Duration (1)lt10yrs, (2)ge10yrs	3 (0.1)	85 (0.1)	1.55 (0.48 - 5.04)
soc5d 29 106 Duration Category	2	Physicians and Surgeons - Duration (1)lt10yrs, (2)ge10yrs	3 (0.1)	141 (0.2)	0.88 (0.27 - 2.80)
soc5d 29 112 Ever	-	Therapists - Ever	22 (1.1)	659 (0.9)	1.21 (0.78 - 1.88)
soc5d 29 112 Duration Category	1	Therapists - Duration (1)lt10yrs, (2)ge10yrs	9 (0.4)	243 (0.3)	1.44 (0.73 - 2.85)
soc5d 29 112 Duration Category	2	Therapists - Duration (1)lt10yrs, (2)ge10yrs	13 (0.6)	411 (0.6)	1.10 (0.62 - 1.93)
soc5d 29 114 Ever	-	Registered Nurses - Ever	134 (6.5)	5582 (7.5)	0.85 (0.71 - 1.02)
soc5d 29 114 Duration Category	1	Registered Nurses - Duration (1)lt10yrs, (2)ge10yrs	32 (1.5)	1482 (2.0)	0.78 (0.54 - 1.12)
soc5d 29 114 Duration Category	2	Registered Nurses - Duration (1)lt10yrs, (2)ge10yrs	102 (4.9)	4060 (5.4)	0.89 (0.72 - 1.09)
soc5d 29 117 Ever	-	Nurse Practitioners - Ever	3 (0.1)	55 (0.1)	1.69 (0.51 - 5.64)
soc5d 29 201 Ever	-	Clinical Laboratory Technologists and Technicians - Ever	26 (1.3)	974 (1.3)	1.00 (0.67 - 1.50)
soc5d 29 201 Duration Category	1	Clinical Laboratory Technologists and Technicians - Duration (1)lt10yrs, (2)ge10yrs	16 (0.8)	449 (0.6)	1.25 (0.75 - 2.09)
soc5d 29 201 Duration Category	2	Clinical Laboratory Technologists and Technicians - Duration (1)lt10yrs, (2)ge10yrs	10 (0.5)	519 (0.7)	0.78 (0.41 - 1.47)
soc5d 29 202 Ever	-	Dental Hygienists - Ever	4 (0.2)	208 (0.3)	0.71 (0.26 - 1.94)
soc5d 29 203 Ever	-	Diagnostic Related Technologists and Technicians - Ever	10 (0.5)	245 (0.3)	1.34 (0.70 - 2.58)
soc5d 29 203 Duration Category	1	Diagnostic Related Technologists and Technicians - Duration (1)lt10yrs, (2)ge10yrs	4 (0.2)	111 (0.1)	1.09 (0.39 - 3.04)
soc5d 29 203 Duration Category	2	Diagnostic Related Technologists and Technicians - Duration (1)lt10yrs, (2)ge10yrs	6 (0.3)	132 (0.2)	1.63 (0.70 - 3.77)
soc5d 29 205 Ever	-	Health Practitioner Support Technologists and Technicians - Ever	7 (0.3)	152 (0.2)	1.82 (0.83 - 3.99)
soc5d 29 206 Ever	-	Licensed Practical and Licensed Vocational Nurses - Ever	27 (1.3)	944 (1.3)	1.11 (0.75 - 1.65)
soc5d 29 206 Duration Category	1	Licensed Practical and Licensed Vocational Nurses - Duration (1)lt10yrs, (2)ge10yrs	9 (0.4)	337 (0.5)	1.05 (0.53 - 2.07)
soc5d 29 206 Duration Category	2	Licensed Practical and Licensed Vocational Nurses - Duration (1)lt10yrs, (2)ge10yrs	17 (0.8)	596 (0.8)	1.10 (0.67 - 1.81)

soc5d 29 209 Ever	-	Miscellaneous Health Technologists and Technicians - Ever	4 (0.2)	71 (0.1)	2.45 (0.87 - 6.92)
soc5d 31 101 Ever	-	Nursing Psychiatric and Home Health Aides - Ever	34 (1.6)	1706 (2.3)	0.78 (0.55 - 1.11)
soc5d 31 101 Duration Category	1	Nursing Psychiatric and Home Health Aides - Duration (1)lt10yrs, (2)ge10yrs	18 (0.9)	1046 (1.4)	0.68 (0.42 - 1.10)
soc5d 31 101 Duration Category	2	Nursing Psychiatric and Home Health Aides - Duration (1)lt10yrs, (2)ge10yrs	14 (0.7)	631 (0.8)	0.84 (0.49 - 1.45)
soc5d 31 909 Ever	-	Miscellaneous Healthcare Support Occupations - Ever	38 (1.8)	1361 (1.8)	1.04 (0.75 - 1.45)
soc5d 31 909 Duration Category	1	Miscellaneous Healthcare Support Occupations - Duration (1)lt10yrs, (2)ge10yrs	22 (1.1)	801 (1.1)	1.01 (0.66 - 1.56)
soc5d 31 909 Duration Category	2	Miscellaneous Healthcare Support Occupations - Duration (1)lt10yrs, (2)ge10yrs	16 (0.8)	544 (0.7)	1.12 (0.67 - 1.87)
soc5d 33 301 Ever	-	Bailiffs Correctional Officers and Jailers - Ever	4 (0.2)	49 (0.1)	2.01 (0.69 - 5.84)
soc5d 33 305 Ever	-	Police Officers - Ever	4 (0.2)	88 (0.1)	1.33 (0.47 - 3.76)
soc5d 33 909 Ever	-	Miscellaneous Protective Service Workers - Ever	6 (0.3)	185 (0.2)	1.14 (0.50 - 2.64)
soc5d 35 101 Ever	-	Supervisors of Food Preparation and Serving Workers - Ever	9 (0.4)	350 (0.5)	0.92 (0.47 - 1.80)
soc5d 35 101 Duration Category	1	Supervisors of Food Preparation and Serving Workers - Duration (1)lt10yrs, (2)ge10yrs	3 (0.1)	175 (0.2)	0.70 (0.22 - 2.23)
soc5d 35 101 Duration Category	2	Supervisors of Food Preparation and Serving Workers - Duration (1)lt10yrs, (2)ge10yrs	6 (0.3)	171 (0.2)	1.10 (0.48 - 2.53)
soc5d 35 201 Ever	-	Cooks - Ever	18 (0.9)	914 (1.2)	0.81 (0.50 - 1.31)
soc5d 35 201 Duration Category	1	Cooks - Duration (1)lt10yrs, (2)ge10yrs	6 (0.3)	536 (0.7)	0.43 (0.19 - 0.96)
soc5d 35 201 Duration Category	2	Cooks - Duration (1)lt10yrs, (2)ge10yrs	12 (0.6)	357 (0.5)	1.53 (0.84 - 2.77)
soc5d 35 301 Ever	-	Bartenders - Ever	8 (0.4)	136 (0.2)	1.34 (0.64 - 2.81)
soc5d 35 301 Duration Category	1	Bartenders - Duration (1)lt10yrs, (2)ge10yrs	5 (0.2)	64 (0.1)	1.81 (0.70 - 4.65)
soc5d 35 301 Duration Category	2	Bartenders - Duration (1)lt10yrs, (2)ge10yrs	3 (0.1)	70 (0.1)	0.96 (0.29 - 3.12)
soc5d 35 302 Ever	-	Fast Food and Counter Workers - Ever	9 (0.4)	276 (0.4)	1.28 (0.65 - 2.53)
soc5d 35 303 Ever	-	Waiters and Waitresses - Ever	81 (3.9)	2034 (2.7)	1.18 (0.94 - 1.50)
soc5d 35 303 Duration Category	1	Waiters and Waitresses - Duration (1)lt10yrs, (2)ge10yrs	53 (2.6)	1373 (1.8)	1.25 (0.94 - 1.66)
soc5d 35 303 Duration Category	2	Waiters and Waitresses - Duration (1)lt10yrs, (2)ge10yrs	27 (1.3)	620 (0.8)	1.10 (0.74 - 1.64)
soc5d 35 304 Ever	-	Food Servers Nonrestaurant - Ever	4 (0.2)	120 (0.2)	1.42 (0.51 - 3.95)
soc5d 35 901 Ever	-	Dining Room and Cafeteria Attendants and Bartender Helpers - Ever	7 (0.3)	259 (0.3)	1.25 (0.58 - 2.70)
soc5d 35 903 Ever	-	Hosts and Hostesses Restaurant Lounge and Coffee Shop - Ever	10 (0.5)	195 (0.3)	1.64 (0.85 - 3.18)
soc5d 37 201 Ever	-	Building Cleaning Workers - Ever	26 (1.3)	1371 (1.8)	0.92 (0.61 - 1.38)
soc5d 37 201 Duration Category	1	Building Cleaning Workers - Duration (1)lt10yrs, (2)ge10yrs	16 (0.8)	736 (1.0)	1.03 (0.62 - 1.71)
soc5d 37 201 Duration Category	2	Building Cleaning Workers - Duration (1)lt10yrs, (2)ge10yrs	8 (0.4)	595 (0.8)	0.68 (0.33 - 1.38)

soc5d 39 202 Ever	-	Nonfarm Animal Caretakers - Ever	4 (0.2)	53 (0.1)	2.34 (0.81 - 6.81)
soc5d 39 301 Ever	-	Gaming Services Workers - Ever	4 (0.2)	72 (0.1)	1.12 (0.39 - 3.20)
soc5d 39 501 Ever	-	Barbers Hairdressers Hairstylists and Cosmetologists - Ever	31 (1.5)	964 (1.3)	1.18 (0.81 - 1.71)
soc5d 39 501 Duration Category	1	Barbers Hairdressers Hairstylists and Cosmetologists - Duration (1)lt10yrs, (2)ge10yrs	9 (0.4)	343 (0.5)	1.08 (0.55 - 2.13)
soc5d 39 501 Duration Category	2	Barbers Hairdressers Hairstylists and Cosmetologists - Duration (1)lt10yrs, (2)ge10yrs	21 (1.0)	599 (0.8)	1.20 (0.77 - 1.89)
soc5d 39 701 Ever	-	Tour and Travel Guides - Ever	6 (0.3)	137 (0.2)	1.88 (0.81 - 4.33)
soc5d 39 901 Ever	-	Childcare Workers - Ever	33 (1.6)	1192 (1.6)	1.34 (0.94 - 1.91)
soc5d 39 901 Duration Category	1	Childcare Workers - Duration (1)lt10yrs, (2)ge10yrs	26 (1.3)	808 (1.1)	1.51 (1.01 - 2.26)
soc5d 39 901 Duration Category	2	Childcare Workers - Duration (1)lt10yrs, (2)ge10yrs	6 (0.3)	345 (0.5)	0.90 (0.40 - 2.05)
soc5d 39 902 Ever	-	Personal Care Aides - Ever	9 (0.4)	377 (0.5)	1.04 (0.53 - 2.06)
soc5d 39 902 Duration Category	1	Personal Care Aides - Duration (1)lt10yrs, (2)ge10yrs	6 (0.3)	263 (0.4)	0.99 (0.43 - 2.27)
soc5d 39 902 Duration Category	2	Personal Care Aides - Duration (1)lt10yrs, (2)ge10yrs	3 (0.1)	101 (0.1)	1.31 (0.40 - 4.29)
soc5d 39 903 Ever	-	Recreation and Fitness Workers - Ever	22 (1.1)	545 (0.7)	1.51 (0.97 - 2.34)
soc5d 39 903 Duration Category	1	Recreation and Fitness Workers - Duration (1)lt10yrs, (2)ge10yrs	17 (0.8)	400 (0.5)	1.53 (0.93 - 2.52)
soc5d 39 903 Duration Category	2	Recreation and Fitness Workers - Duration (1)lt10yrs, (2)ge10yrs	5 (0.2)	135 (0.2)	1.55 (0.62 - 3.86)
soc5d 41 101 Ever	-	FirstLine Supervisors of Sales Workers - Ever	76 (3.7)	2178 (2.9)	1.10 (0.86 - 1.39)
soc5d 41 101 Duration Category	1	FirstLine Supervisors of Sales Workers - Duration (1)lt10yrs, (2)ge10yrs	36 (1.7)	1047 (1.4)	1.06 (0.76 - 1.50)
soc5d 41 101 Duration Category	2	FirstLine Supervisors of Sales Workers - Duration (1)lt10yrs, (2)ge10yrs	40 (1.9)	1109 (1.5)	1.16 (0.84 - 1.61)
soc5d 41 201 Ever	-	Cashiers - Ever	52 (2.5)	1726 (2.3)	1.06 (0.79 - 1.41)
soc5d 41 201 Duration Category	1	Cashiers - Duration (1)lt10yrs, (2)ge10yrs	37 (1.8)	1233 (1.7)	1.03 (0.74 - 1.45)
soc5d 41 201 Duration Category	2	Cashiers - Duration (1)lt10yrs, (2)ge10yrs	15 (0.7)	471 (0.6)	1.18 (0.69 - 2.01)
soc5d 41 203 Ever	-	Retail Salespersons - Ever	199 (9.6)	7175 (9.6)	1.01 (0.87 - 1.17)
soc5d 41 203 Duration Category	1	Retail Salespersons - Duration (1)lt10yrs, (2)ge10yrs	142 (6.8)	5130 (6.9)	1.00 (0.84 - 1.20)
soc5d 41 203 Duration Category	2	Retail Salespersons - Duration (1)lt10yrs, (2)ge10yrs	56 (2.7)	1916 (2.6)	1.07 (0.81 - 1.41)
soc5d 41 301 Ever	-	Advertising Sales Agents - Ever	6 (0.3)	197 (0.3)	0.84 (0.37 - 1.93)
soc5d 41 302 Ever	-	Insurance Sales Agents - Ever	13 (0.6)	477 (0.6)	0.90 (0.51 - 1.58)
soc5d 41 302 Duration Category	1	Insurance Sales Agents - Duration (1)lt10yrs, (2)ge10yrs	5 (0.2)	258 (0.3)	0.64 (0.26 - 1.56)
soc5d 41 302 Duration Category	2	Insurance Sales Agents - Duration (1)lt10yrs, (2)ge10yrs	8 (0.4)	213 (0.3)	1.29 (0.62 - 2.67)
soc5d 41 303 Ever	-	Securities Commodities and Financial Services Sales Agents - Ever	3 (0.1)	159 (0.2)	0.54 (0.17 - 1.72)
soc5d 41 304 Ever	-	Travel Agents - Ever	17 (0.8)	573 (0.8)	0.88 (0.54 - 1.45)
soc5d 41 304 Duration Category	1	Travel Agents - Duration (1)lt10yrs, (2)ge10yrs	8 (0.4)	265 (0.4)	1.02 (0.50 - 2.09)

soc5d 41 304 Duration Category	2	Travel Agents - Duration (1)lt10yrs, (2)ge10yrs	9 (0.4)	304 (0.4)	0.80 (0.41 - 1.57)
soc5d 41 309 Ever	-	Miscellaneous Sales Representatives Services - Ever	11 (0.5)	458 (0.6)	0.75 (0.41 - 1.39)
soc5d 41 309 Duration Category	1	Miscellaneous Sales Representatives Services - Duration (1)lt10yrs, (2)ge10yrs	6 (0.3)	296 (0.4)	0.68 (0.30 - 1.55)
soc5d 41 309 Duration Category	2	Miscellaneous Sales Representatives Services - Duration (1)lt10yrs, (2)ge10yrs	5 (0.2)	156 (0.2)	0.88 (0.35 - 2.19)
soc5d 41 401 Ever	-	Sales Representatives Wholesale and Manufacturing - Ever	26 (1.3)	533 (0.7)	1.52 (1.01 - 2.29)
soc5d 41 401 Duration Category	1	Sales Representatives Wholesale and Manufacturing - Duration (1)lt10yrs, (2)ge10yrs	16 (0.8)	310 (0.4)	1.70 (1.01 - 2.86)
soc5d 41 401 Duration Category	2	Sales Representatives Wholesale and Manufacturing - Duration (1)lt10yrs, (2)ge10yrs	10 (0.5)	218 (0.3)	1.32 (0.69 - 2.55)
soc5d 41 901 Ever	-	Models Demonstrators and Product Promoters - Ever	13 (0.6)	383 (0.5)	1.18 (0.67 - 2.08)
soc5d 41 901 Duration Category	1	Models Demonstrators and Product Promoters - Duration (1)lt10yrs, (2)ge10yrs	10 (0.5)	286 (0.4)	1.20 (0.63 - 2.30)
soc5d 41 901 Duration Category	2	Models Demonstrators and Product Promoters - Duration (1)lt10yrs, (2)ge10yrs	3 (0.1)	92 (0.1)	1.16 (0.36 - 3.75)
soc5d 41 902 Ever	-	Real Estate Brokers and Sales Agents - Ever	72 (3.5)	2231 (3.0)	0.96 (0.75 - 1.23)
soc5d 41 902 Duration Category	1	Real Estate Brokers and Sales Agents - Duration (1)lt10yrs, (2)ge10yrs	26 (1.3)	954 (1.3)	0.80 (0.53 - 1.19)
soc5d 41 902 Duration Category	2	Real Estate Brokers and Sales Agents - Duration (1)lt10yrs, (2)ge10yrs	46 (2.2)	1249 (1.7)	1.11 (0.82 - 1.51)
soc5d 41 909 Ever	-	Miscellaneous Sales and Related Workers - Ever	19 (0.9)	492 (0.7)	1.34 (0.83 - 2.15)
soc5d 41 909 Duration Category	1	Miscellaneous Sales and Related Workers - Duration (1)lt10yrs, (2)ge10yrs	11 (0.5)	315 (0.4)	1.15 (0.62 - 2.13)
soc5d 41 909 Duration Category	2	Miscellaneous Sales and Related Workers - Duration (1)lt10yrs, (2)ge10yrs	7 (0.3)	170 (0.2)	1.58 (0.73 - 3.45)
soc5d 43 101 Ever	-	FirstLine Supervisors of Office and Administrative Support Workers - Ever	63 (3.0)	1833 (2.5)	1.06 (0.82 - 1.38)
soc5d 43 101 Duration Category	1	FirstLine Supervisors of Office and Administrative Support Workers - Duration (1)lt10yrs,	31 (1.5)	875 (1.2)	1.06 (0.73 - 1.53)
soc5d 43 101 Duration Category	2	FirstLine Supervisors of Office and Administrative Support Workers - Duration (1)lt10yrs,	32 (1.5)	935 (1.3)	1.09 (0.76 - 1.57)
soc5d 43 201 Ever	-	Switchboard Operators Including Answering Service - Ever	13 (0.6)	327 (0.4)	1.25 (0.70 - 2.21)
soc5d 43 201 Duration Category	1	Switchboard Operators Including Answering Service - Duration (1)lt10yrs, (2)ge10yrs	9 (0.4)	226 (0.3)	1.22 (0.61 - 2.42)
soc5d 43 201 Duration Category	2	Switchboard Operators Including Answering Service - Duration (1)lt10yrs, (2)ge10yrs	4 (0.2)	95 (0.1)	1.37 (0.49 - 3.84)
soc5d 43 202 Ever	-	Telephone Operators - Ever	27 (1.3)	1182 (1.6)	0.80 (0.54 - 1.19)
soc5d 43 202 Duration Category	1	Telephone Operators - Duration (1)lt10yrs, (2)ge10yrs	23 (1.1)	863 (1.2)	0.97 (0.63 - 1.48)
soc5d 43 202 Duration Category	2	Telephone Operators - Duration (1)lt10yrs, (2)ge10yrs	4 (0.2)	310 (0.4)	0.42 (0.15 - 1.13)
soc5d 43 301 Ever	-	Bill and Account Collectors - Ever	3 (0.1)	88 (0.1)	1.27 (0.39 - 4.17)
soc5d 43 302 Ever	-	Billing and Posting Clerks - Ever	20 (1.0)	606 (0.8)	1.18 (0.75 - 1.87)

soc5d 43 302 Duration Category	1	Billing and Posting Clerks - Duration (1)lt10yrs, (2)ge10yrs	17 (0.8)	444 (0.6)	1.33 (0.81 - 2.20)
soc5d 43 302 Duration Category	2	Billing and Posting Clerks - Duration (1)lt10yrs, (2)ge10yrs	3 (0.1)	159 (0.2)	0.72 (0.23 - 2.30)
soc5d 43 303 Ever	-	Bookkeeping Accounting and Auditing Clerks - Ever	162 (7.8)	4755 (6.4)	1.18 (1.00 - 1.40)
soc5d 43 303 Duration Category	1	Bookkeeping Accounting and Auditing Clerks - Duration (1)lt10yrs, (2)ge10yrs	95 (4.6)	2605 (3.5)	1.27 (1.02 - 1.57)
soc5d 43 303 Duration Category	2	Bookkeeping Accounting and Auditing Clerks - Duration (1)lt10yrs, (2)ge10yrs	66 (3.2)	2113 (2.8)	1.09 (0.84 - 1.40)
soc5d 43 305 Ever	-	Payroll and Timekeeping Clerks - Ever	18 (0.9)	522 (0.7)	1.17 (0.72 - 1.90)
soc5d 43 305 Duration Category	1	Payroll and Timekeeping Clerks - Duration (1)lt10yrs, (2)ge10yrs	15 (0.7)	354 (0.5)	1.39 (0.81 - 2.36)
soc5d 43 305 Duration Category	2	Payroll and Timekeeping Clerks - Duration (1)lt10yrs, (2)ge10yrs	3 (0.1)	163 (0.2)	0.70 (0.22 - 2.22)
soc5d 43 307 Ever	-	Tellers - Ever	27 (1.3)	1018 (1.4)	0.88 (0.59 - 1.30)
soc5d 43 307 Duration Category	1	Tellers - Duration (1)lt10yrs, (2)ge10yrs	21 (1.0)	690 (0.9)	0.99 (0.64 - 1.56)
soc5d 43 307 Duration Category	2	Tellers - Duration (1)lt10yrs, (2)ge10yrs	6 (0.3)	318 (0.4)	0.64 (0.28 - 1.44)
soc5d 43 309 Ever	-	Miscellaneous Financial Clerks - Ever	8 (0.4)	277 (0.4)	1.14 (0.56 - 2.35)
soc5d 43 309 Duration Category	1	Miscellaneous Financial Clerks - Duration (1)lt10yrs, (2)ge10yrs	4 (0.2)	176 (0.2)	0.88 (0.32 - 2.42)
soc5d 43 309 Duration Category	2	Miscellaneous Financial Clerks - Duration (1)lt10yrs, (2)ge10yrs	4 (0.2)	96 (0.1)	1.72 (0.62 - 4.81)
soc5d 43 403 Ever	-	Court Municipal and License Clerks - Ever	4 (0.2)	152 (0.2)	0.92 (0.34 - 2.54)
soc5d 43 404 Ever	-	Credit Authorizers Checkers and Clerks - Ever	6 (0.3)	210 (0.3)	0.90 (0.39 - 2.05)
soc5d 43 404 Duration Category	1	Credit Authorizers Checkers and Clerks - Duration (1)lt10yrs, (2)ge10yrs	3 (0.1)	163 (0.2)	0.62 (0.20 - 1.98)
soc5d 43 404 Duration Category	2	Credit Authorizers Checkers and Clerks - Duration (1)lt10yrs, (2)ge10yrs	3 (0.1)	45 (0.1)	1.66 (0.49 - 5.61)
soc5d 43 405 Ever	-	Customer Service Representatives - Ever	24 (1.2)	870 (1.2)	0.94 (0.62 - 1.43)
soc5d 43 405 Duration Category	1	Customer Service Representatives - Duration (1)lt10yrs, (2)ge10yrs	11 (0.5)	487 (0.7)	0.79 (0.43 - 1.46)
soc5d 43 405 Duration Category	2	Customer Service Representatives - Duration (1)lt10yrs, (2)ge10yrs	12 (0.6)	378 (0.5)	1.04 (0.58 - 1.88)
soc5d 43 406 Ever	-	Eligibility Interviewers Government Programs - Ever	4 (0.2)	139 (0.2)	1.11 (0.40 - 3.07)
soc5d 43 407 Ever	-	File Clerks - Ever	14 (0.7)	563 (0.8)	0.88 (0.51 - 1.51)
soc5d 43 407 Duration Category	1	File Clerks - Duration (1)lt10yrs, (2)ge10yrs	9 (0.4)	437 (0.6)	0.70 (0.36 - 1.38)
soc5d 43 407 Duration Category	2	File Clerks - Duration (1)lt10yrs, (2)ge10yrs	5 (0.2)	112 (0.1)	1.72 (0.68 - 4.34)
soc5d 43 411 Ever	-	Interviewers Except Eligibility and Loan - Ever	17 (0.8)	555 (0.7)	0.99 (0.60 - 1.63)
soc5d 43 411 Duration Category	1	Interviewers Except Eligibility and Loan - Duration (1)lt10yrs, (2)ge10yrs	10 (0.5)	371 (0.5)	0.88 (0.46 - 1.68)
soc5d 43 411 Duration Category	2	Interviewers Except Eligibility and Loan - Duration (1)lt10yrs, (2)ge10yrs	7 (0.3)	170 (0.2)	1.31 (0.60 - 2.86)
soc5d 43 412 Ever	-	Library Assistants Clerical - Ever	18 (0.9)	663 (0.9)	1.06 (0.66 - 1.71)

soc5d 43 412 Duration Category	1	Library Assistants Clerical - Duration (1)lt10yrs, (2)ge10yrs	10 (0.5)	433 (0.6)	0.89 (0.47 - 1.69)
soc5d 43 412 Duration Category	2	Library Assistants Clerical - Duration (1)lt10yrs, (2)ge10yrs	8 (0.4)	222 (0.3)	1.43 (0.69 - 2.94)
soc5d 43 415 Ever	-	Order Clerks - Ever	4 (0.2)	206 (0.3)	0.67 (0.25 - 1.83)
soc5d 43 416 Ever	-	Human Resources Assistants Except Payroll and Timekeeping - Ever	6 (0.3)	407 (0.5)	0.50 (0.22 - 1.14)
soc5d 43 417 Ever	-	Receptionists and Information Clerks - Ever	58 (2.8)	2124 (2.8)	0.99 (0.75 - 1.29)
soc5d 43 417 Duration Category	1	Receptionists and Information Clerks - Duration (1)lt10yrs, (2)ge10yrs	47 (2.3)	1583 (2.1)	1.07 (0.80 - 1.45)
soc5d 43 417 Duration Category	2	Receptionists and Information Clerks - Duration (1)lt10yrs, (2)ge10yrs	11 (0.5)	508 (0.7)	0.79 (0.43 - 1.44)
soc5d 43 418 Ever	-	Reservation and Transportation Ticket Agents and Travel Clerks - Ever	7 (0.3)	302 (0.4)	0.67 (0.31 - 1.44)
soc5d 43 418 Duration Category	1	Reservation and Transportation Ticket Agents and Travel Clerks - Duration (1)lt10yrs, (2)g	3 (0.1)	189 (0.3)	0.42 (0.13 - 1.32)
soc5d 43 418 Duration Category	2	Reservation and Transportation Ticket Agents and Travel Clerks - Duration (1)lt10yrs, (2)g	4 (0.2)	113 (0.2)	1.22 (0.44 - 3.39)
soc5d 43 419 Ever	-	Miscellaneous Information and Record Clerks - Ever	4 (0.2)	194 (0.3)	0.83 (0.30 - 2.28)
soc5d 43 502 Ever	-	Couriers and Messengers - Ever	3 (0.1)	92 (0.1)	1.29 (0.40 - 4.15)
soc5d 43 505 Ever	-	Postal Service Workers - Ever	16 (0.8)	499 (0.7)	1.18 (0.71 - 1.98)
soc5d 43 505 Duration Category	1	Postal Service Workers - Duration (1)lt10yrs, (2)ge10yrs	9 (0.4)	213 (0.3)	1.50 (0.75 - 3.00)
soc5d 43 505 Duration Category	2	Postal Service Workers - Duration (1)lt10yrs, (2)ge10yrs	7 (0.3)	281 (0.4)	0.94 (0.44 - 2.04)
soc5d 43 506 Ever	-	Production Planning and Expediting Clerks - Ever	5 (0.2)	230 (0.3)	0.69 (0.28 - 1.70)
soc5d 43 508 Ever	-	Stock Clerks and Order Fillers - Ever	20 (1.0)	637 (0.9)	1.10 (0.70 - 1.75)
soc5d 43 508 Duration Category	1	Stock Clerks and Order Fillers - Duration (1)lt10yrs, (2)ge10yrs	12 (0.6)	428 (0.6)	0.97 (0.54 - 1.74)
soc5d 43 508 Duration Category	2	Stock Clerks and Order Fillers - Duration (1)lt10yrs, (2)ge10yrs	8 (0.4)	200 (0.3)	1.46 (0.70 - 3.05)
soc5d 43 511 Ever	-	Weighers Measurers Checkers and Samplers Recordkeeping - Ever	4 (0.2)	81 (0.1)	1.93 (0.68 - 5.52)
soc5d 43 601 Ever	-	Secretaries and Administrative Assistants - Ever	438 (21.1)	15993 (21.4)	0.93 (0.83 - 1.04)
soc5d 43 601 Duration Category	1	Secretaries and Administrative Assistants - Duration (1)lt10yrs, (2)ge10yrs	221 (10.7)	7859 (10.5)	0.97 (0.84 - 1.12)
soc5d 43 601 Duration Category	2	Secretaries and Administrative Assistants - Duration (1)lt10yrs, (2)ge10yrs	216 (10.4)	8016 (10.7)	0.90 (0.77 - 1.04)
soc5d 43 901 Ever	-	Computer Operators - Ever	18 (0.9)	485 (0.6)	1.31 (0.81 - 2.13)
soc5d 43 901 Duration Category	1	Computer Operators - Duration (1)lt10yrs, (2)ge10yrs	10 (0.5)	291 (0.4)	1.26 (0.66 - 2.41)
soc5d 43 901 Duration Category	2	Computer Operators - Duration (1)lt10yrs, (2)ge10yrs	8 (0.4)	186 (0.2)	1.42 (0.68 - 2.95)
soc5d 43 902 Ever	-	Data Entry and Information Processing Workers - Ever	66 (3.2)	2380 (3.2)	1.02 (0.79 - 1.32)
soc5d 43 902 Duration Category	1	Data Entry and Information Processing Workers - Duration (1)lt10yrs, (2)ge10yrs	50 (2.4)	1722 (2.3)	1.10 (0.82 - 1.48)

soc5d 43 902 Duration Category	2	Data Entry and Information Processing Workers - Duration (1)lt10yrs, (2)ge10yrs	15 (0.7)	636 (0.9)	0.79 (0.47 - 1.34)
soc5d 43 904 Ever	-	Insurance Claims and Policy Processing Clerks - Ever	15 (0.7)	397 (0.5)	1.24 (0.73 - 2.11)
soc5d 43 904 Duration Category	1	Insurance Claims and Policy Processing Clerks - Duration (1)lt10yrs, (2)ge10yrs	11 (0.5)	282 (0.4)	1.29 (0.70 - 2.41)
soc5d 43 904 Duration Category	2	Insurance Claims and Policy Processing Clerks - Duration (1)lt10yrs, (2)ge10yrs	4 (0.2)	113 (0.2)	1.11 (0.40 - 3.10)
soc5d 43 905 Ever	-	Mail Clerks and Mail Machine Operators Except Postal Service - Ever	4 (0.2)	153 (0.2)	0.97 (0.35 - 2.69)
soc5d 43 906 Ever	-	Office Clerks General - Ever	184 (8.9)	7137 (9.6)	0.93 (0.79 - 1.09)
soc5d 43 906 Duration Category	1	Office Clerks General - Duration (1)lt10yrs, (2)ge10yrs	117 (5.6)	4613 (6.2)	0.93 (0.76 - 1.12)
soc5d 43 906 Duration Category	2	Office Clerks General - Duration (1)lt10yrs, (2)ge10yrs	66 (3.2)	2432 (3.3)	0.95 (0.74 - 1.23)
soc5d 43 908 Ever	-	Proofreaders and Copy Markers - Ever	4 (0.2)	111 (0.1)	1.25 (0.45 - 3.45)
soc5d 43 911 Ever	-	Statistical Assistants - Ever	5 (0.2)	158 (0.2)	1.22 (0.49 - 3.03)
soc5d 43 919 Ever	-	Miscellaneous Office and Administrative Support Workers - Ever	38 (1.8)	1557 (2.1)	0.91 (0.66 - 1.27)
soc5d 43 919 Duration Category	1	Miscellaneous Office and Administrative Support Workers - Duration (1)lt10yrs, (2)ge10yrs	26 (1.3)	1068 (1.4)	0.90 (0.60 - 1.33)
soc5d 43 919 Duration Category	2	Miscellaneous Office and Administrative Support Workers - Duration (1)lt10yrs, (2)ge10yrs	10 (0.5)	467 (0.6)	0.81 (0.43 - 1.54)
soc5d 45 204 Ever	-	Graders and Sorters Agricultural Products - Ever	3 (0.1)	48 (0.1)	2.41 (0.70 - 8.28)
soc5d 45 209 Ever	-	Miscellaneous Agricultural Workers - Ever	8 (0.4)	176 (0.2)	1.85 (0.89 - 3.86)
soc5d 47 214 Ever	-	Painters and Paperhangers - Ever	3 (0.1)	102 (0.1)	1.12 (0.34 - 3.64)
soc5d 47 221 Ever	-	Sheet Metal Workers - Ever	3 (0.1)	9 (0.0)	15.36 (3.42 - 68.92)
soc5d 51 101 Ever	-	FirstLine Supervisors of Production and Operating Workers - Ever	3 (0.1)	220 (0.3)	0.47 (0.15 - 1.47)
soc5d 51 202 Ever	-	Electrical Electronics and Electromechanical Assemblers - Ever	8 (0.4)	300 (0.4)	0.89 (0.43 - 1.83)
soc5d 51 209 Ever	-	Miscellaneous Assemblers and Fabricators - Ever	19 (0.9)	501 (0.7)	1.48 (0.92 - 2.37)
soc5d 51 209 Duration Category	1	Miscellaneous Assemblers and Fabricators - Duration (1)lt10yrs, (2)ge10yrs	14 (0.7)	332 (0.4)	1.60 (0.92 - 2.78)
soc5d 51 209 Duration Category	2	Miscellaneous Assemblers and Fabricators - Duration (1)lt10yrs, (2)ge10yrs	5 (0.2)	162 (0.2)	1.25 (0.50 - 3.11)
soc5d 51 301 Ever	-	Bakers - Ever	3 (0.1)	119 (0.2)	1.09 (0.34 - 3.55)
soc5d 51 412 Ever	-	Welding Soldering and Brazing Workers - Ever	5 (0.2)	106 (0.1)	1.85 (0.73 - 4.68)
soc5d 51 419 Ever	-	Miscellaneous Metal Workers and Plastic Workers - Ever	8 (0.4)	168 (0.2)	1.61 (0.77 - 3.37)
soc5d 51 419 Duration Category	1	Miscellaneous Metal Workers and Plastic Workers - Duration (1)lt10yrs, (2)ge10yrs	4 (0.2)	103 (0.1)	1.22 (0.44 - 3.40)
soc5d 51 419 Duration Category	2	Miscellaneous Metal Workers and Plastic Workers - Duration (1)lt10yrs, (2)ge10yrs	4 (0.2)	64 (0.1)	2.39 (0.82 - 6.98)
soc5d 51 511 Ever	-	Printing Workers - Ever	13 (0.6)	292 (0.4)	1.39 (0.78 - 2.47)

soc5d 51 511 Duration Category	1	Printing Workers - Duration (1)lt10yrs, (2)ge10yrs	8 (0.4)	149 (0.2)	1.62 (0.78 - 3.40)
soc5d 51 511 Duration Category	2	Printing Workers - Duration (1)lt10yrs, (2)ge10yrs	5 (0.2)	136 (0.2)	1.17 (0.47 - 2.94)
soc5d 51 602 Ever	-	Pressers Textile Garment and Related Materials - Ever	3 (0.1)	126 (0.2)	0.96 (0.30 - 3.08)
soc5d 51 603 Ever	-	Sewing Machine Operators - Ever	14 (0.7)	745 (1.0)	0.88 (0.51 - 1.51)
soc5d 51 603 Duration Category	1	Sewing Machine Operators - Duration (1)lt10yrs, (2)ge10yrs	7 (0.3)	381 (0.5)	0.85 (0.40 - 1.83)
soc5d 51 603 Duration Category	2	Sewing Machine Operators - Duration (1)lt10yrs, (2)ge10yrs	7 (0.3)	349 (0.5)	0.92 (0.43 - 1.99)
soc5d 51 605 Ever	-	Tailors Dressmakers and Sewers - Ever	6 (0.3)	273 (0.4)	1.01 (0.44 - 2.31)
soc5d 51 606 Ever	-	Textile Machine Setters Operators and Tenders - Ever	4 (0.2)	106 (0.1)	1.51 (0.54 - 4.25)
soc5d 51 609 Ever	-	Miscellaneous Textile Apparel and Furnishings Workers - Ever	4 (0.2)	101 (0.1)	1.68 (0.60 - 4.71)
soc5d 51 906 Ever	-	Inspectors Testers Sorters Samplers and Weighers - Ever	13 (0.6)	582 (0.8)	0.89 (0.51 - 1.56)
soc5d 51 906 Duration Category	1	Inspectors Testers Sorters Samplers and Weighers - Duration (1)lt10yrs, (2)ge10yrs	3 (0.1)	343 (0.5)	0.33 (0.10 - 1.03)
soc5d 51 906 Duration Category	2	Inspectors Testers Sorters Samplers and Weighers - Duration (1)lt10yrs, (2)ge10yrs	9 (0.4)	228 (0.3)	1.66 (0.83 - 3.29)
soc5d 51 911 Ever	-	Packaging and Filling Machine Operators and Tenders - Ever	10 (0.5)	290 (0.4)	1.34 (0.70 - 2.57)
soc5d 51 911 Duration Category	1	Packaging and Filling Machine Operators and Tenders - Duration (1)lt10yrs, (2)ge10yrs	7 (0.3)	219 (0.3)	1.29 (0.59 - 2.80)
soc5d 51 911 Duration Category	2	Packaging and Filling Machine Operators and Tenders - Duration (1)lt10yrs, (2)ge10yrs	3 (0.1)	63 (0.1)	1.75 (0.52 - 5.81)
soc5d 51 915 Ever	-	Photographic Process Workers and Processing Machine Operators - Ever	3 (0.1)	117 (0.2)	0.88 (0.27 - 2.83)
soc5d 51 919 Ever	-	Miscellaneous Production Workers - Ever	24 (1.2)	922 (1.2)	0.97 (0.64 - 1.48)
soc5d 51 919 Duration Category	1	Miscellaneous Production Workers - Duration (1)lt10yrs, (2)ge10yrs	17 (0.8)	591 (0.8)	1.07 (0.65 - 1.75)
soc5d 51 919 Duration Category	2	Miscellaneous Production Workers - Duration (1)lt10yrs, (2)ge10yrs	7 (0.3)	308 (0.4)	0.87 (0.40 - 1.87)
soc5d 53 203 Ever	-	Flight Attendants - Ever	10 (0.5)	315 (0.4)	0.98 (0.51 - 1.86)
soc5d 53 302 Ever	-	Bus Drivers - Ever	8 (0.4)	368 (0.5)	0.80 (0.39 - 1.62)
soc5d 53 302 Duration Category	1	Bus Drivers - Duration (1)lt10yrs, (2)ge10yrs	3 (0.1)	165 (0.2)	0.61 (0.19 - 1.94)
soc5d 53 302 Duration Category	2	Bus Drivers - Duration (1)lt10yrs, (2)ge10yrs	4 (0.2)	198 (0.3)	0.79 (0.29 - 2.15)
soc5d 53 303 Ever	-	Driver Sales Workers and Truck Drivers - Ever	10 (0.5)	155 (0.2)	1.64 (0.84 - 3.20)
soc5d 53 303 Duration Category	1	Driver Sales Workers and Truck Drivers - Duration (1)lt10yrs, (2)ge10yrs	7 (0.3)	106 (0.1)	1.72 (0.77 - 3.83)
soc5d 53 303 Duration Category	2	Driver Sales Workers and Truck Drivers - Duration (1)lt10yrs, (2)ge10yrs	3 (0.1)	48 (0.1)	1.54 (0.46 - 5.21)
soc5d 53 706 Ever	-	Laborers and Material Movers Hand - Ever	15 (0.7)	490 (0.7)	1.20 (0.71 - 2.05)
soc5d 53 706 Duration Category	1	Laborers and Material Movers Hand - Duration (1)lt10yrs, (2)ge10yrs	10 (0.5)	341 (0.5)	1.17 (0.61 - 2.24)

soc5d 53 706 Duration Category	2	Laborers and Material Movers Hand - Duration (1)lt10yrs, (2)ge10yrs	5 (0.2)	139 (0.2)	1.34 (0.53 - 3.35)

*Odds ratios (ORs) and 95% confidence intervals (CIs) were derived from logistic regression models adjusted for covariates: age at enrollment, smoking status, pack years of smoking, worked with smoker, lived with smoker as a child, lived with smoker after age 18, alcohol intake, U. S. region, education, Hispanic, race, and cancer in first-degree relative.

Table includes all occupations at the 2-digit, 3-digit, and 5-digit SOC levels with at least 3 cases
